# Effect of Botanical Extracts on the Growth and Nutritional Quality of Field-Grown White Head Cabbage (*Brassica oleracea* var. *capitata*)

**DOI:** 10.3390/molecules26071992

**Published:** 2021-04-01

**Authors:** Katarzyna Godlewska, Paweł Pacyga, Izabela Michalak, Anita Biesiada, Antoni Szumny, Natalia Pachura, Urszula Piszcz

**Affiliations:** 1 Department of Horticulture, Faculty of Life Sciences and Technology, Wrocław University of Environmental and Life Sciences, 50-363 Wrocław, Poland; anita.biesiada@upwr.edu.pl; 2 Department of Energy Technologies, Turbines, and Modeling of Heat-Flow Processes, Faculty of Mechanical and Power Engineering, Wrocław University of Science and Technology, 50-370 Wrocław, Poland; pawel.pacyga@pwr.edu.pl; 3 Department of Advanced Material Technologies, Faculty of Chemistry, Wrocław University of Science and Technology, 50-372 Wrocław, Poland; izabela.michalak@pwr.edu.pl; 4 Department of Chemistry, Faculty of Biotechnology and Food Science, Wrocław University of Environmental and Life Sciences, 50-375 Wrocław, Poland; antoni.szumny@upwr.edu.pl (A.S.); natalia.pachura@upwr.edu.pl (N.P.); 5 Department of Plant Nutrition, Faculty of Life Sciences and Technology, Wrocław University of Environmental and Life Sciences, 50-357 Wrocław, Poland; urszula.piszcz@upwr.edu.pl

**Keywords:** higher plants, extraction, bioactive compounds, white head cabbage, yield, nutritional quality, sustainable food production

## Abstract

Nutraceuticals and functional foods are gaining more attention amongst consumers interested in nutritious food. The consumption of foodstuffs with a high content of phytochemicals has been proven to provide various health benefits. The application of biostimulants is a potential strategy to fortify cultivated plants with beneficial bioactive compounds. Nevertheless, it has not yet been established whether the proposed higher plants (St. John’s wort, giant goldenrod, common dandelion, red clover, nettle, and valerian) are appropriate for the production of potential bio-products enhancing the nutritional value of white cabbage. Therefore, this research examines the impact of botanical extracts on the growth and nutritional quality of cabbage grown under field conditions. Two extraction methods were used for the production of water-based bio-products, namely: ultrasound-assisted extraction and mechanical homogenisation. Bio-products were applied as foliar sprays to evaluate their impact on total yield, dry weight, photosynthetic pigments, polyphenols, antioxidant activity, vitamin C, nitrates, micro- and macroelements, volatile compounds, fatty acids, sterols, and sugars. Botanical extracts showed different effects on the examined parameters. The best results in terms of physiological and biochemical properties of cabbage were obtained for extracts from common dandelion, valerian, nettle, and giant goldenrod. When enriched with nutrients, vegetables can constitute a valuable component of functional food.

## 1. Introduction

Certain dietary components that are not necessarily required for an individual’s existence may affect the quality of life by altering a single or several physiologic processes. This ability depends on various factors, such as interactions with other constituents, the consumer’s physiologic state, behaviour pattern, and genetics [1]. People are confident that food may influence the risk of developing different kinds of diseases [1,2,3,4,5]. Chronic diseases, which may be triggered by multiple factors (including unhealthy food), constitute one of the major public health problems in developing countries [6]. The growing demand for health-promoting food, containing physiologically active compounds, as well as the availability of novel food, can be expected in the future [1]. One of the promising and potential strategies for enhancing health and immunity, maintaining well-being, preventing and lowering the risk of chronic diseases are functional products, namely, functional food, food supplements, and nutraceuticals [6,7]. Moreover, growing interest in functional products is emerging due to increased health care costs and scientific discoveries [1].

Natural or processed food that, beyond providing energy and basic nutrients (fatty acids, proteins, dietary fibre, carbohydrates, vitamins, minerals, antioxidants, prebiotics, probiotics, symbiotics, and phytochemicals), has an additional beneficial impact on health, physical performance, or state of mind is called functional food [1,2,4,5,8,9]. The consumption of this type of food strengthens the immune system, averts diseases (prevents from heart, brain, and kidney-related diseases), decreases cholesterol levels, reduces lactose intolerance, remission of Crohn’s disease, relieves diarrhoea, inhibits cancer cell proliferation, helps to recover from physical and mental disorders, and can also delay the ageing process [10,11]. Vegetables and fruits are rich sources of several bioactive compounds and constitute the simplest form of functional food [7]. They could be freely consumed as a part of an everyday life diet [5,8,12]. 

On the other hand, nutraceuticals are concentrated bioactive compounds refined from food and are the foremost products used in healthcare [7,9,12,13,14]. They can be found in various pharmaceutical forms—liquids, pills, capsules, tablets, powders, potions, solutions, and vials [2,7,9]. They are defined as any food (or a part thereof) with benefits for human health additional to the basic nutritional value found in foodstuffs [14,15]. The term “nutraceutical” results from two words—“nutrient” and “pharmaceutical” [10,14,15,16,17]. The interest in nutraceuticals amongst the scientific community, consumers, and food manufacturers has been growing steadily in recent years [2] due to their potential nutritional and therapeutic effects as well as safeness [14]. The deciphering mechanism of their action will contribute to the development of the next generation of therapeutic agents [18]. A broad range of phytochemicals, diverse compounds produced by plants [13], including, for example, organosulfur compounds (e.g., allyl sulfides), glucosinolates, monoterpenes, terpenoids (tetraterpenoids (e.g., carotenoids) and triterpenoids (e.g., limonoids)), phytosterols, carbohydrates (e.g., oligosaccharides), peptides, polyphenols (anthocyanins, ellagic acid, flavonoids, flavanones, isoflavones, proanthocyanidins, and resveratrol), stilbenoids, polyunsaturated fatty acids, and vitamins can act as nutraceuticals [2,5,13,17,19]. They exhibit a wide spectrum of biological activities and play a role in numerous processes (e.g., antioxidant defences, cell proliferation, gene expression, and safeguarding of mitochondrial integrity). They can be used to postpone the ageing process, treat and prevent diseases like cancer, coronary heart and neurodegenerative diseases, diabetes, obesity, inflammation, Alzheimer’s and Parkinson’s diseases, high blood pressure, and spasmodic disorders [5,12,14,18,20]. It is considered that there are 4000 phytochemicals present in vegetables, fruits, and grains, thus their rational consumption can provide these valuable compounds [13]. 

Vegetables grown in sustainable and environmentally friendly systems can be a source of functional food and/or nutraceuticals. Biostimulants of plant growth could be used to increase the content of active compounds in cultivated plants. The agricultural production, apart from increasing the crops yields, should focus on enhancing their nutritional quality, particularly during unfavourable environmental conditions [21], along with the improvement of resource use efficiency (water and fertilisers) [22]. One of the most promising, cutting-edge, sustainable solutions is the use of biostimulants rich in bioavailable, bioactive compounds [23,24], which stimulate different physiological processes in plants and, as a result, provide potential benefits to growth and development, and have nutritional value, health-promoting potential, or stress response benefits [22,25]. According to The European Biostimulant Industry Council (EBIC) “Plant biostimulant means a material which contains substance(s) and/or microorganisms whose function when applied to plants or the rhizosphere is to stimulate natural processes to benefit nutrient uptake, nutrient efficiency, tolerance to abiotic stress, and/or crop quality, independently of its nutrient content.” [26]. Initially, biostimulants were applied in organic production, but nowadays, they are also used in conventional and integrated crop production [22]. The most widely used bio-products are based on amino acids, algal extracts, and humic compounds [27]. 

Quite recently, considerable attention has been paid to the use of new raw materials for the production of biostimulants. Thereupon, this study was undertaken to evaluate the impact of botanical extracts obtained through ultrasound-assisted extraction (UAE) and mechanical homogenisation (MH) on white head cabbage. Our results cast a new light on the possibility of using higher plants commonly occurring in Europe (e.g., St. John’s wort, giant goldenrod, common dandelion, red clover, nettle, and valerian) for the production of potential biostimulants. As a model plant, white head cabbage has been chosen as it is one of the most widely cultivated crops in the world.

## 2. Materials and Methods

The experiments were carried out in compliance with the methods given in detail in our previous work [28]. The concise methodology is described below.

### 2.1. The Tested Raw Materials and Botanical Extract Production

The following raw materials were used for the production of biostimulants: St. John’s wort (*Hypericum perforatum* L.; herb) (marked as Hp H), giant goldenrod (*Solidago gigantea* Ait.; leaf; Sg L), common dandelion (*Taraxacum officinale* L. Weber ex F.H. Wigg; flower, leaf; To F and To L), red clover (*Trifolium pratense* L.; flower; Tp F), nettle (*Urtica dioica* L.; leaf; Ur L), and valerian (*Valeriana officinalis* L.; root; Vo R). In our preliminary research, we have selected 26 different biomasses and assessed their potential biostimulatory effects [29,30]. On the basis of obtained results, we chose the best 7 for further field trial tests. To investigate the impact of properties of the final bio-products, two techniques were selected: ultrasound-assisted extraction and mechanical shearing combined with sonic energy. Before production of extracts, the raw materials were dried (50 °C), ground, sieved (500 μm mesh size), and mixed with deionised water (1:20 *w*/*v*). Next, the mixture was soaked (30 min) and sonicated (30 min) (for UAE) or homogenised (1 min, 28,000 rpm) (for MH), and centrifuged (10 min, 4500 rpm). The obtained supernatant constituted a 100% extract. The final formulation consisted of the active ingredient (extract, 0.5% *w*/*v*), an adjuvant (Protector, 0.02% *w*/*v*), an antioxidant agent (L-ascorbic acid, 0.15% *w*/*v*), a preservative (potassium sorbate, 0.1% *w*/*v*), and water (up to 100%).

### 2.2. The Field Trials

The field trials on white head cabbage (*Brassica oleracea* var. *capitata*) were conducted under Polish climate conditions (Figure S1) in randomised complete blocks in three replicates. Fine clay soil was pre-plant fertilised with Hydrocomplex Yara Mila (250 kg·ha^−1^) and ammonium saltpetre (330 kg·ha^−1^), and top-dressed with ammonium saltpetre (two times, 165 kg·ha^−1^ each). Seeds (cultivar ‘Socrates F1’, Syngenta) were sown on 9 May in a glasshouse, and seedlings were planted out on 22 May to the soil on 8 June with spacing 60 cm × 60 cm (plot size: 7.2 m^2^; 20 plants per plot), and harvested on 6 November 2018. The botanical extracts were applied three times on sunny, windless days in the morning at a dose of 1000 L·ha^−1^ on: 12 July, 22 July, and 1 August 2018. The control groups were sprayed with water (C), formulation without active ingredient—plant extract (CF), and commercial biostimulant (CB). Typical cultivation treatments (e.g., regular mechanical weeding, irrigation) were conducted. Furthermore, insecticides: Decis Mega 50 EW (Bayer, Leverkusen, Germany), Karate Zeon 050 CS (Syngenta, Basel, Switzerland), and Bi 58 Top 400 EC (BASF, Ludwigshafen, Germany) were applied according to the manufacturer’s recommendations. Samples were collected for analyses 7 days after the second spraying and at the end of the experiment.

### 2.3. The Photosynthetic Pigments, Greenness Index of the Leaves, and Leaf Colour

The analyses of the contents of chlorophyll *a* + *b* and carotenoids were made using fresh outer leaves collected 7 days after the second spraying. A pestle and mortar were used to homogenise the samples (0.4 g) with a few drops of acetone (80%) and a pinch of sand with calcium carbonate. The homogenised pulp was filtered and moved to a volumetric flask (50 mL) and filled with acetone (80%). The absorbances were measured in 4 replicates at pigment-specific peak wavelengths (663, 645, and 470 nm) using a spectrophotometer (HACH DR1900, Berlin, Germany). The greenness indexes were assessed in the fresh leaves in 16 replicates in each experimental group, using a SPAD 502 Plus Chlorophyll Meter (Konica Minolta, Osaka, Japan), and the colours of the leaves in 16 replicates using MiniScan (Hunter Lab EZ, Reston, VA, USA). The Hunter *L*, *a*, *b* colour space is a three-dimensional rectangular colour space based on Opponent-Colours Theory (*L* (lightness) axis—0 is black, 50 is middle grey, 100 is white; *a* (red-green) axis—positive values are red, 0 is neutral, negative values are green; *b* (blue-yellow) axis—positive values are yellow, 0 is neutral, negative values are blue) [29,30,31].

### 2.4. Vitamin C

The content of the vitamin C was evaluated in 4 replicates in fresh leaves (~8 g) and heads (~12 g). Samples were homogenised in oxalic acid (200 mL, 2%), filtrated, and obtained solutions (10 mL) were titrated with Tillmans’ reagent until a light pinkish colour appeared and was maintained for at least 30 s [32,33,34].

### 2.5. Total Phenolic Compounds

The total phenolic compound (TPC) content was estimated in fresh leaves and heads (~2 g). Samples were mixed with aqueous methanol (20 mL, 80%) in tubes, sonicated (15 min) and centrifuged (10 min, 4500 rpm). The Folin–Ciocalteu’s phenol reagent (0.2 mL) and distilled water (2 mL) were added to the supernatants (0.1 mL), mixed and kept in the dark (3 min). Next, sodium carbonate (1 mL, 20%) was added and left in the dark (1 h), and the absorbance (765 nm) was measured. Analyses were performed in 4 replicates [29,30,35].

### 2.6. The Antioxidant Activity (DPPH, ABTS, and FRAP)

The antioxidant activities were evaluated in ten-fold diluted supernatants prepared for TPC analyses. The analyses were made in 4 replicates.

For the DPPH assay, ethanol (1.5 mL) and DPPH solution (0.5 mL) were added to the supernatants (0.5 mL), properly mixed and incubated in the dark at room temperature (10 min). The absorbance was measured at a wavelength of 517 nm [29,30,36].

For the ABTS assay, a diluted ABTS solution (3 mL) was added to the supernatants (30 μL) and left in the dark (6 min). The absorbance was measured at a wavelength of 734 nm [29,30,37,38].

For the FRAP assay, a FRAP reagent (3 mL) was added to the supernatant (1 mL), and the mixture was kept to react (10 min). The absorbance was measured at a wavelength of 593 nm [29,30,39].

### 2.7. Nitrates

The analyses of nitrate content were conducted in dried and ground samples (0.4 g), which were shaken (30 min, 150 rpm) with acetic acid (100 mL, 2%) and activated carbon (0.5–1.0 g). The readings were made in filtrated solution using an ionometer (Thermo 5 Star Orion, Beverly, MA, USA) with an ion-selective electrode [33,40].

### 2.8. Macroelements, Microelements, and Toxic Elements

The analyses of the content of macroelements (P, K, Ca, and Mg), microelements (Mn, Fe, Cu, Zn, and Ni), and heavy metals (Cd and Pb) were performed using dried and ground cabbage heads. Samples were mineralised (450 °C, 8 h) in an oven (CZYLOK, Jastrzębie-Zdrój, Poland). The obtained ash was digested (65% HNO_3_) and evaporated on a heating plate (110 °C, 6 h), and then dissolved (1 M HNO_3_) and transferred to a flask. The content of P was assessed using a colourimetric method (400 nm) with molybdate and ammonium metavanadate (Cecil CE 2011 photometer; Cambridge, UK); the content of K, Ca, Mg, Mn, Fe, Cu, Zn, Cd, Pb, and Ni were measured using atomic absorption spectrophotometry (ASA) (Varian Spectra AA 220/FS instrument, Mulgrave, Australia); the content of S was measured by using the oxidation of sulphur on the basis of the turbidity of the solution of sulphate content precipitating as barium sulphate (Cecil CE 2011 photometer; Cambridge, UK); the content of N was measured using the Kjeldahl method [41,42,43].

### 2.9. Volatile Compounds

The volatile compound chromatographic analyses (GC-MS; GCMS QP 2020, Shimadzu, Kyoto, Japan) were made using frozen heads (55 g) in 3 replicates. Samples were placed in round-bottom flasks (250 mL) and boiled with distilled water (100 mL) in a heating mantle. As an internal standard (IS), 1 mL of cyclohexane containing 1 mg of 2-undecanone was used. A distillation process (50 min) was performed using a Deryng apparatus [44]. 

### 2.10. Fatty Acids

The lipid fraction was prepared from dried cabbage heads (300 mg) which were macerated with chloroform (5 mL), filtered, and evaporated. The extracted nonpolar lipid fraction (25 mg) was saponified (5 min at 65 °C) with 0.5 M KOH/MeOH solution (2 mL) and subjected to methylation (10 min at 65 °C) by adding 14% (*v*/*v*) BF_3_/MeOH (2 mL). Next, distilled water (5 mL) was added, and the methyl esters of fatty acids were extracted with hexane (10 mL). The solution was washed with 10% sodium bicarbonate (10 mL) and dried over anhydrous sodium sulphate. The organic phase was evaporated under reduced pressure and dissolved in hexane (200 µL). The profiles of fatty acid methyl esters were analysed by gas chromatograph coupled with a mass spectrometer (GC-MS) (Shimadzu GCMS QP 2020, Shimadzu, Kyoto, Japan) in 3 replicates [45,46,47].

### 2.11. Sterols

The sterol determination procedure was carried out in accordance with the methods presented by Abidi [48] with modifications. The analyses were made using dried cabbage heads (300 mg). Samples were macerated with chloroform (5 mL), and the sterol profile was assessed using the method of derivatisation with N,O-Bis (trimethylsililyl)trifluoroacetamide (BSTFA) silylation using GC-MS (Shimadzu QP 2020, Shimadzu, Kyoto, Japan). The mixture was filtered and evaporated on a vacuum evaporator under reduced pressure. In the next step, pyridine (500 µL) and BSTFA (50 µL) were added, and the mixture was transferred to a vial and heated for 15 min at 70 °C. Separation was achieved using a Zebron ZB-5 capillary column (30 m, 0.25 mm, 0.25 μm; Phenomenex, Torrance, CA, USA). The GC-MS analysis was performed according to the following parameters: scan mode with a mass range from 40 to 1050 *m*/*z* in the electronic impact (EI) mode at 70 eV mode with the 10 scan s^−1^ mode. Analyses were conducted using helium as a carrier gas at a flow rate of 1 mL min^−1^ in a split ratio of 1:20 along with the following program: (a) 100 °C for 1 min; (b) a rate of 2 °C min^−1^ from 100 to 190 °C; (c) a rate of 5 °C min^−1^ from 190 to 300 °C. The injector was maintained at 280 °C. Compounds were identified using two different analytical methods to compare the retention times with those of authentic chemicals (Supelco C7-C40 Saturated Alkanes Standard, Sigma-Aldrich, St. Louis, MI, USA), and the mass spectra were obtained with the available library data (Willey NIST 17, match index >90%, Hoboken, NJ, USA).

### 2.12. Glucosinolates

Freeze-dried samples of 1 g were extracted with 70% methanol (10 mL; 30 min, 70 °C). During the extraction process, samples were shaken on a vortex every 3 min, then filtered and centrifuged (10 min, 15,000 rpm). The supernatant was separated, methanol was removed from the mixture by evaporation on a vacuum evaporator. Next, samples were prepared by dissolving in water (1 mL), and the LC-MS analysis was performed using reversed-phase high-performance liquid chromatography (HPLC Shimadzu Prominence-i LC-2030C, Kyoto, Japan) equipped with a PDA detector coupled to a triple quadrupole mass spectrometer (Shimadzu LCMS-8045). Glycosides were separated using the following mobile phase: water with 0.1% TFA (eluent A) and acetonitrile with 0.1% TFA (eluent B). The flow rate was set at 0.25 mL·min^−1^, and the gradient was as follows: starting at 1% solvent B for 3 min, then reaching 20% up to 20 min, 30% up to 23 min, and 0.1% B at 35 min. Separation was obtained on a Kinetex C18 100A column (100 × 3 mm, 2.6 µm particle size, Phenomenex, Germany). Singrin and neoglucobrassicin were used as external standards for the analysis [49].

### 2.13. Sugars

Sugar content was assessed according to the standard procedure of the Polish Committee for Standardisation [50] used for the analysis of fruit and vegetable products (a Lane and Eynon general volumetric method). Fragmented and defrosted samples of cabbage heads (~20 g) were placed in volumetric flasks (250 mL). Next, distilled water (~120 mL) was added, and flasks were boiled in a water bath (30 min). After that time, cold water was added, and flasks were left to cool. In the next step, Carrez I (5 mL) and Carrez II (5 mL) solutions were used for the deproteinisation, and flasks were filled up with water, well shaken, and filtered. For the reducing sugars, Fehling’s solution (10 mL) and sucrose solution (8 mL) was added to the filtrates (10 mL), heated, and boiled (2 min). Next, methylene blue (~3 drops) was added, and solutions were titrated until the colour turned brick red. For the total sugars, concentrated HCl (5 mL) was added to the filtrates (50 mL) in a volumetric flask (100 mL). Solutions were heated in a water bath until reaching the temperature of 70 °C, then were taken out, hydrolysed (3 min), and chilled to 20 °C within 1 min. Next, NaOH (30%) was added to neutralise, and flasks were filled up with water. Solutions were poured into a beaker to settle the sediments. Next, Fehling’s solution (10 mL) and sucrose solution (8 mL) were added to the collected solution (10 mL), and the procedure was repeated as in the case of reducing sugars. Sugar content was expressed in percentages (%) of fresh weight.

### 2.14. Statistical Analyses

The statistical analyses were performed using the STATISTICA software ver. 13.3 (TIBCO Software Inc., Tulsa, OK, USA). The differences that were statistically significant (*p* < 0.05) between the groups treated with the botanical extracts and the control group (C) were marked in tables and figures with “a”, between the formulation (CF) with “b” and between commercial biostimulant (CB) with “c”.

## 3. Results

### 3.1. Total Yield, Fresh and Dry Weight of White Head Cabbage and Outer Leaves

In the majority of cases, the application of botanical extracts did not have a statistically significant effect on the yield of cabbage heads (Table 1). The bio-product based on Hp H (UAE) was an exception—an increase was observed by 13.3% in comparison to the control group treated with water (C), whereas the use of Sg L MH and To F UAE caused a significant decrease—13.7% and 12.4%, respectively when compared to C. On the other hand, it can be seen that the obtained formulations promoted the growth of outer leaves, especially Sg L UAE and Hp H UAE—in these groups, the total yield was higher by 16.4% and 10.2% than in C. The least stimulating activity was noted after the use of Ur L UAE—9.0% and 16.3% less than in C and CB, respectively. During the harvest, the heads were divided into two size ranges: lighter than 1.2 kg (marked as head < 1.2 kg) and heavier than 1.2 kg (head > 1.2 kg). As shown in Table 1, the botanical extracts tended to foster the growth of smaller heads, in particular: Tp F UAE (50% and 106% more than in C and CB, respectively) as well as Vo R UAE (37.9% and 89.6% more than in C and CB, respectively), Hp H MH (36.4% and 87.5% more than in C and CB, respectively), To L UAE (36.4% and 87.5% more than in C and CB, respectively), and Ur L UAE (33.3% and 83.3% more than in C and CB, respectively). The lowest weight was found in the groups sprayed with commercial biostimulant (27.3% less than in C) and Vo R MH (33.3% less than in C). It is discernible that the aforementioned bio-products, Vo R UAE, Ur L UAE, and Hp H MH, stimulated the fresh weight of outer leaves to the greatest extent, by 37.5%, 35.2%, and 23.9% more than in C, respectively, and by 49.4%, 46.9%, and 34.6% more than in CB, respectively. The application of To L UAE (26.1% and 19.8% less than in C and CB, respectively) and To F MH (23.9% and 17.3% less than in C and CB, respectively) was the least favourable. Moreover, the foliar spraying with the extracts did not lead to an increase in the fresh weight in the group of heads heavier than 1.2 kg (for instance, the application of Sg L MH lowered the weight by 14.6% and 18.6% in relation to C and CB, respectively) except for the use of Hp H UAE that resulted in 8.8% and 3.6% growth in relation to C and CB, respectively. 

The dry weight was assessed in outer leaves collected seven days after the second spraying and in heads after the harvest (Table 1). As indicated in the figures, statistically significant differences were observed in the first term of sample collection. The botanical extracts obtained from To F (MH) and Ur L (UAE) increased the dry weight (DW) by 10.2% and 7.5% in comparison to C, respectively, and by 7.8% and 5.2% when compared to CB, respectively. The least stimulation was noted after the use of Hp H UAE—7.6% and 9.6% less than in C and CB, respectively. As in the case of the total yield, the dry weight of the collected heads was not altered by the majority of extracts. Similarly, Hp H UAE and additionally Vo R UAE had the highest impact on dry weight—3.7% and 4.3% more than in C and 7.1% and 7.7% more than in CB, respectively. It can be noticed that Ur L MH had the slightest effect on the dry weight (9.7% and 6.8% less than in C and CB, respectively).

To achieve higher cabbage head yields, an extract based on Hp H can be recommended for further research.

### 3.2. The Photosynthetic Pigments, Greenness Index of the Leaves, and Leaf Colour

To investigate the impact of botanical extracts on the colour of leaves, samples were collected seven days after the second spraying and subjected to analyses using three separate methods. We found that the obtained formulations significantly affected the examined parameters. Table 2 details the data on the content of chlorophyll *a* + *b* in leaves measured spectrophotometrically. It was observed that To L MH increased the amount to the greatest extent (46.0% and 24.0% more than in C and CB, respectively), followed by To F MH (31.5% and 11.6% more than in C and CB, respectively), Hp H MH (30.6% and 11% more than in C and CB, respectively), To L UAE (28.2% and 8.9% more than in C and CB, respectively), Sg L UAE (25.8% and 6.8% more than in C and CB, respectively) and Sg L MH (25% and 6.2% more than in C and CB, respectively). The largest decrease was noted in plants sprayed with To F UAE (6.5% more than in C and 9.6% less than in CB) and Vo R MH (9.7% more than in C and 6.8% less than in CB). Overall, a similar trend was seen in the case of carotenoids (Table 2) but mostly without statistically significant differences—To L MH exerted the highest influence on the content on these pigments (21.8% and 19.2% more than in C and CB, respectively), whilst Vo R MH had the lowest (4.4% and 6.4% less than in C and CB, respectively).

It is notable from Table 2 that the examined formulations, in general, had no significant relationship with the *L*, *a*, and *b* values evaluated in the control group (C). A few exceptions included, for example, the *L* value: To F MH (15.2% more than in C), Tp F MH (12% more than in C) and Sg L MH (11.9% more than in C), for *a* value: Tp F MH (12.1% more than in C), and for *b* value: Tp F MH (20.7% more than in C). It was apparent that the foliar spraying with commercial biostimulant had the most beneficial effect on the tested values, e.g., for the *L* value, it was 14.5% more, for the *a* value, it was 14.3% more, and for the *b* value, it was 24.6% more, in comparison to C.

The measurements of the SPAD values are presented in Table 2. The results showed that botanical extracts positively impacted the greenness index of leaves, for instance, when treated with To F MH (12.4% and 21.8% more than in C and CB, respectively), Sg L MH (10% and 19.2% more than in C and CB, respectively), Ur L MH (7.7% and 16.7% more than in C and CB, respectively), Vo R MH (7.6% and 16.6% more than in C and CB, respectively), and To L UAE (7.4% more and 16.4% more than in C and CB). The lowest values were obtained for ultrasound-assisted extracts based on Sg L (0.8% less than in C and 7.6% more than in CB) and To F (0.5% and 8.9% more than in C and CB, respectively). 

The use of extracts based on To L, Tp F, To F, and Sg L could be advisable to increase the colour of plant leaves.

### 3.3. Vitamin C

It can be seen from Table 2 that the content of vitamin C in cabbage leaves collected seven days after the second spraying was elevated due to the use of extracts based on higher plants, especially obtained through mechanical homogenisation, e.g., Tp F (52.1% and 25.9% more than in C and CB, respectively), Sg L (31.0% and 8.4% more than in C and CB, respectively), To F (29.2% and 6.9% more than in C and CB, respectively), but also through ultrasound-assisted extraction, e.g., To F (30.0% and 7.3% more than in C and CB, respectively). However, in most cases, bio-products produced by UAE exerted a lower impact on the content of the examined vitamin than those by MH. For instance, Vo R and Sg L reduced the amount by 0.8% and 0.3% in comparison to C and by 17.9% and 17.5% in relation to CB, respectively. As shown in Table 2, there was no correlation between the application of extracts and the content of vitamin C. The To L UAE extract was the only one that increased its content by 28.2% when compared with the control group sprayed with water. It could be observed that the application of commercial biostimulant had the greatest impact on the reduction in the vitamin C content.

### 3.4. Total Phenolic Compounds and Antioxidant Activity (DPPH, ABTS, and FRAP)

The influence of botanical extracts on the content of total phenolic compounds (TPC) in leaves collected seven days after the second spraying and in cabbage heads is depicted in Table 3. Comparing the results, it could be seen that ultrasound-assisted extraction was a better method for the production of potential biostimulants than mechanical homogenisation to achieve crop yields with higher TPC content. Nonetheless, in both terms of cabbage collection, the application of the majority of the botanical extracts did not increase the TPC content. For both leaves and heads, the highest content of TPC was noted after the application of Vo R UAE (in the first term: 39.7% and 136% more than in C and CB, respectively; in the second term: 34.6% and 60.3% more than in C and CB, respectively) and Sg L UAE (in the first term: 6.1% and 79.7% more than in C and CB, respectively; in the second term: 6.9% and 27.4% more than in C and CB, respectively). The lowest values were in the groups treated with Ur L MH (in the first term: 47.7% and 11.5% less than in C and CB, respectively; in the second term: 29.7% and 16.3% less than in C and CB, respectively) and Hp H UAE (in the second term: 30.2% and 17.3% less than in C and CB, respectively). The application of commercial biostimulant also did not have a positive effect on this parameter. 

The influence of the examined extracts use on the antioxidant activity of cabbage measured using DPPH assay is shown in Table 3. The conducted analyses revealed that the following extracts: To L UAE (21.2% and 12.9% more than in C and CB, respectively), Vo R UAE (16.8% and 8.8% more than in C and CB, respectively), Sg L UAE (10.2% and 2.7% more than in C and CB, respectively), Hp H UAE (5.1% more than in C and 2.0% less than in CB, respectively), and Vo R MH (2.9% more than in C and 4.1% less than in CB, respectively) had the greatest effect on the antioxidant activity in samples collected in the first term. In the second term, only three of them: Vo R MH (48.9% and 379% more than in C and CB, respectively), Sg L UAE (40% and 350% more than in C and CB, respectively), and Hp H UAE (15.6% and 271% more than in C and CB, respectively) demonstrated the highest stimulating activity. The lowest values were determined for: Tp F UAE (in the first term: 30.7% and 35.4% less than in C and CB, respectively; in the second term: 37.8% less than in C and 100% more than in CB, respectively), Ur L UAE (in the first term: 31.4% and 36.1% less than in C and CB, respectively), Tp F MH (in the first term: 33.6% and 38.1% less than in C and CB, respectively; in the second term: 51.1% less than in C and 57.1% more than in CB, respectively), and Vo R UAE (in the second term: 57.8% less than in C and 35.7% more than in CB, respectively). 

The use of bio-products, as well as commercial biostimulant, exhibited differential effects on the antioxidant activity measured via ABTS assay, namely, the activity significantly increased after the second spraying whilst after the third spraying, it was lowered (Table 3) with reference to the control group (C). It was also noticed that in the first term of samples collection, the higher activity was determined for the application of extracts obtained through mechanical homogenisation than ultrasound-assisted extraction, whereas such a correlation was not observed in the second term. In outer leaves, the activity was the highest after the application of Sg L MH (67.4% and 39.2% more than in C and CB, respectively), Ur L UAE (56.6% and 30.2% more than in C and CB, respectively), Hp H MH (48.5% and 23.5% more than in C and CB, respectively), and Hp H UAE (45.6% and 21.2% more than in C and CB, respectively), whereas in cabbage heads it was the highest for Hp H MH (47.7% and 121% more than in C and CB, respectively). The lowest antioxidant activity was revealed for the following extracts: To F UAE (15.8% more than in C and 3.7% less than in CB) and Vo R UAE (18.7% more than in C and 1.3% less than in CB) measured in outer leaves, and for To F UAE (72.1% and 58.2% less than in C and CB) as determined in heads.

In the case of the antioxidant activity measured via FRAP assay, there was a discernible relationship between the use of botanical extracts and the examined parameter, which is illustrated in Table 3. In the first term of samples collection, the highest activity was noted in the groups treated with Hp H UAE (33.9% and 62% more than in C and CB, respectively), Tp F UAE (28.3% and 55.3% more than in C and CB, respectively), Sg L UAE (27.3% and 54.0% more than in C and CB, respectively), and Sg L MH (26% and 52.2% more than in C and CB, respectively). The lowest activity was observed after the use of Ur L MH (16.1% less than in C and 1.5% more than in CB), Hp H MH (14.5% less than in C and 3.5% more than in CB), and To L MH (13.8% less than in C and 4.3% more than in CB). It was seen that ultrasound-assisted extraction improved antioxidant activity of outer leaves compared to mechanical homogenisation, whilst a similar correlation was not observed in the case of analyses of heads. In the second term, the highest activity was noted for the following bio-products: Tp F UAE (37.4% and 10.5% more than in C and CB, respectively) and Vo R MH (36.6% and 9.8% more than in C and CB, respectively), and the lowest was noted for To L MH (26% and 40.5% less than in C and CB, respectively). 

Based on the results obtained, the following raw materials: Vo R (TPC, DPPH, and FRAP assay), Sg L (TPC, DPPH, ABTS, and FRAP assay), Hp H (DPPH, ABTS, and FRAP assay), To L (DPPH assay), Ur L (ABTS assay), and Tp F (FRAP assay) could be recommended for further investigation as TPC and antioxidant activity enhancing products.

### 3.5. Nitrates

It was apparent that the tested botanical extracts tend to diminish the content of nitrates in leaves taken for analyses seven days after the second spraying and tend to increase the accumulation of nitrates in cabbage heads (Table 4). Analogous tendencies could be observed in the groups sprayed with a commercial product. In the first term of samples collection, extracts obtained through mechanical homogenisation based on Sg L, To L, and Vo R decreased their content to the greatest extent, namely, by 58%, 53.4%, and 50%, respectively, in comparison to the control group treated with water and by 25.6%, 17.6%, and 11.4%, respectively, in relation to commercial biostimulant. The highest amounts of nitrates were observed after the use of To L UAE (0.3% less than in C and 76.5% more than in CB), Sg L UAE (16.7% less than in C and 47.4% more than in CB), Ur L MH (19.5% less than in C and 42.5% more than in CB), and Tp F UAE (20.6% less than in C and 40.5% more than in CB). In the second term of samples collection, the highest enhancement of nitrates accumulation was noted in the groups sprayed with: Tp F MH (186% and 115% more than in C and CB, respectively), To F UAE (89.9% and 42.7% more than in C and CB, respectively), Sg L MH (84.9% and 38.9% more than in C and CB, respectively), To L MH (75.1% and 31.6% more than in C and CB), and To L UAE (73% and 30% more than in C and CB, respectively) whilst the lowest was noted with Vo R UAE (2.4% more than in C and 23% less than in CB), Ur L MH (22% more than in C and 8.4% less than in CB), and Sg L UAE (26.4% more than in C and 5.1% less than in CB).

The use of bio-products should be adjusted to the needs of specific crops. Further research is required to decipher the exact impact of the examined extracts on nitrate accumulation. From the above findings, it could be seen that Sg L MH and To L MH lowered nitrates content in outer leaves whilst in heads: these products worked conversely. Similar observations could be made for Sg L UAE and Ur L MH—they increased the amount of nitrates in samples from the first term collection but decreased the amount in samples in the second. The only extract that increased their accumulation in both terms was To L UAE. Additionally, the use of Tp F extract should be investigated further, as formulations obtained through ultrasound-assisted extraction increased their content in outer leaves whilst mechanical homogenisation enhanced their content in heads.

### 3.6. Macroelements, Microelements, and Toxic Elements

Generally, the botanical extracts positively influenced the content of macroelements in white head cabbage (Table 4). Only in the case of P and K, their values were lower in all the experimental groups than in the control group treated with water (C) and commercial biostimulant (CB)—with the exception of Ur L MH (P slightly higher—3.1% than in CB). Nitrogen content was higher in the groups treated with the botanical extracts than in C (with the exception of Sg L MH and Tp F MH) and CB, but only for Sg L UAE and To L MH (the best groups). The content of Ca, Mg, and S in cabbage from the experimental groups was higher than in C. The best results for Ca were obtained for To L UAE and Tp F MH (36.1% higher than in C and 27% higher than in CB), for Mg in the group Tp F MH (17.9% higher than in C and 9.8% higher than in CB), for S in the Hp H MH (10.9% higher than in C and slightly higher—1.0% than in CB) and To L MH groups (11.2% higher than in C and slightly higher—1.3% than in CB).

The content of microelements in the cabbage was poorly influenced by examined botanical extracts (Table 5). Iron increased in the groups treated with Sg L UAE (higher by 7.8%), To L MH (higher by 12.2%) and Vo R MH (higher by 14.3%). Only for the last group, the content of Fe was slightly higher than in the group treated with the commercial biostimulant. The content of Cu was higher than in the control group (C) only for To F UAE (by only 1.5%). Plants treated with water, commercial biostimulant and Hp H MH extract had the same content of Mn. The extract produced from Hp H was the best when compared to others tested. The content of Zn in cabbage in the experimental groups was lower than for the commercial biostimulant, but slightly higher for Hp H MH, To F MH, To L MH, Vo R UAE, and Sg L UAE (the best results—increase of 5.3% when compared to C). The application of the botanical extracts increased the content of Ni, Cd, and Pb in cabbage (higher levels than in the control group). 

In summary, the extract produced from common dandelion had the greatest influence on the content of micro and macroelements in the cabbage biomass.

### 3.7. Volatile Compounds

The literature shows that methanethiol, dimethyl disulfide, and dimethyl trisulfide are mostly responsible for objectionable sulfurous aromas and overcooked off-flavours in cruciferous vegetables [51]. In the tested cabbage heads, 53 volatile compounds were identified (Table S1). It was found that three compounds constituted the largest percentage of the whole GC-MS chromatogram area, namely IS, trisulfide (dimethyl-), and tetrasulfide (dimethyl-). The content of ISs, in general, accounted for about 40% of all extracted volatiles compounds and was the most stimulated by the application of Sg L UAE (6.7% and 15.4% more than in C and CB, respectively) and To L UAE (6.0% and 14.6% more than in C and CB, respectively), and the least stimulated by To F UAE (25.1% and 19.0% less than in C and CB, respectively), and Hp H MH (24.5% and 18.4% less than in C and CB, respectively). Then, the trisulfide (dimethyl-) content was about 30% of the total chromatogram area, and the following bio-products To F UAE (11.6% and 11.1% more than in C and CB, respectively), Vo R MH (10.5% and 10.0% more than in C and CB, respectively), and Tp F UAE (9.5% and 9.0% more than in C and CB, respectively) increased its amount to the greatest extent, while Sg L UAE (15.1% and 15.5% less than in C and CB, respectively), Tp F MH (13.7% and 14.1% less than in C and CB, respectively), and To L UAE (12.0% and 12.4% less than in C and CB, respectively) was lowered the most. The third most abundant compound was tetrasulfide (dimethyl-) and accounted for about 15% of the total chromatogram area. The foliar spray with Hp H MH increased its content the most (28.3% and 16.0% more than in C and CB, respectively), while Tp F MH decreased (24.9% and 32.1% less than in C and CB, respectively). It could be seen that the extracts exerted differential effects on the composition of the volatile compounds, and it is difficult to choose one specific bio-product with the best properties.

### 3.8. Fatty Acids

The tested cabbage heads were characterised by a high content of the following fatty acids (methyl ester): hexadecanoic acid, linolenic acid, linoleic acid, 9Z-9-octadecenoic acid (ethyl ester), and octadecanoic acid (Table S2). The content of hexadecanoic acid (methyl ester) was the most elevated in the groups treated with To L MH (34.7% and 11.3% more than in C and CB, respectively), To F MH (27.9% and 5.7% more than in C and CB, respectively), and Tp F MH (22.7% and 1.3% more than in C and CB, respectively), while it was lowered after the use of Vo R UAE (21.4% and 35.1% less than in C and CB, respectively) and Sg L UAE (12.9% and 28.0% less than in C and CB, respectively). 

The amount of linolenic acid (methyl ester) was stimulated to the greatest extent after foliar spraying with Vo R UAE (66.0% and 90.2% more than in C and CB, respectively), To L UAE (55.3% and 78.0% more than in C and CB, respectively), and Sg L UAE (39.2% and 59.5% more than in C and CB, respectively), while to a lesser extent after the application of To L MH (49.8% and 42.5% less than in C and CB, respectively) and Vo R MH (31.3% and 21.3% less than in C and CB, respectively). 

The higher values of linoleic acid (methyl ester) were observed in plants sprayed with Vo R UAE (14.1% and aa.3% more than in C and CB, respectively), Sg L UAE (13.5% and 21.7% more than in C and CB, respectively), and Ur L UAE (10.9% and 18.8% more than in C and CB, respectively), while the lower in cabbages sprayed with To L MH (32.3% and 27.4% less than in C and CB, respectively) and Vo R MH (22.7% and 17.2% less than in C and CB, respectively). 

Concerning the 9Z-9-octadecenoic acid (ethyl ester), its content was the highest in the groups treated with To L MH (43.6% and 50.2% more than in C and CB, respectively) and Vo R MH (39.5% and 46.0% more than in C and CB, respectively), and the lowest in Vo R UAE (40.8% and 38.0% less than in C and CB, respectively) and To L UAE (31.0% and 27.8% less than in C and CB, respectively) groups. Concerning the quantitative effect on the amount of octadecanoic acid (methyl ester), the greatest effect was with the application of To F MH (14.0% and 54.8% more than in C and CB, respectively) and Hp H UAE (7.1% and 45.4% more than in C and CB, respectively), while Vo R UAE (65.0% and 52.5% less than in C and CB, respectively) and To L UAE (60.2% and 45.9% less than in C and CB) less affected its content.

The application of botanical extracts had a significant impact on the composition of fatty acids present in cabbage heads. This suggests that Sg L UAE, To F MH, To L MH, and Vo R UAE could be considered as possible bio-products to modify the composition of fatty acids and to increase the content of the most important molecules.

### 3.9. Sterols

Table 6 shows the percentage contents of two sterols determined in the cabbage heads. The application of botanical extracts did not statistically affect the content of sterols, besides the valerian extract (MH). In this case, the content of campesterol was higher by 10.1% and 11.1% than in C and CB, respectively. However, the content of β-sitosterol was lower (and the lowest in all the experimental groups) by 2.3% and 2.5% than in C and CB, respectively. A similar tendency was observed after the treatment with To F UAE—the content of campesterol increased (9.9% and 10.9% more than in C and CB, respectively) while β-sitosterol was the second-lowest among all the groups (2.3% and 2.5% less than in C and CB, respectively). The lowest values of campesterol (but still higher than in the control groups) were noted after the foliar spraying with Tp F MH (1.2% and 2.2% more than in C and CB, respectively) and Sg L UAE (1.4% and 2.3% more than in C and CB). On the other hand, the highest amounts of β-sitosterol (but still lower than in the control groups) were observed after treatment with Hp H UAE (0.4% and 0.6% less than in C and CB, respectively) and Sg L UAE (0.3% and 0.5% less than in C and CB, respectively).

### 3.10. Glucosinolates

The glucosinolates detected on LC-MS are presented in Table 7. It could be seen that the foliar application with botanical extracts did not have a statistically significant impact on their content. According to the results obtained from chromatographic analyses, the highest percentage content of all detected glucosinolates was neoglucobrassicin (~27%) followed by singrin (~16%), glucobrassicin (~14%), and glucoraphanin (~14%).

### 3.11. Sugars

The data on the content of sugars in cabbage heads are summarised in Table 6. Conducted tests revealed that the botanical extracts, on average, raised the content of reducing sugars (RSGs) but had a smaller impact on the content of total sugars (TSGs). The increase in the amount of RSGs was noticeable, especially after the use of To F MH (49.2% and 39% more than in C and CB, respectively) and Hp H UAE (47.6% and 37.6% more than in C and CB, respectively) whereas a diminution was seen for Vo R UAE (31.4% and 36.1% less than in C and CB, respectively), Tp F UAE (10.5% and 16.6% less than in C and CB, respectively), Ur L MH (5.8% and 12.2% less than in C and CB, respectively), and To F UAE (2.1% more than in C and 4.9% less than in CB). The content of TSGs was the most elevated in the groups treated with Hp H UAE (27.7% and 38.8% more than in C and CB, respectively), To F MH (26.0% and 36.9% more than in C and CB, respectively), and Hp H MH (12.6% and 22.4% more than in C and CB, respectively) and the least with Tp F UAE (15.6% and 8.2% less than in C and CB, respectively), Ur L MH (15.4% and 8% less than in C and CB, respectively,) and To F UAE (13.4% and 5.9% less than in C and CB, respectively). The application of commercial biostimulants slightly enhanced the content of RSGs and reduced of TSGs. In general, mechanical homogenisation promoted an increase in the content of sugars in cabbage heads. Hp H UAE and To F MH could be considered for further research.

## 4. Discussion

Our results cast a new light on the possibility of using different raw materials in the production of potential biostimulants, increasing yield and nutritional composition.

### 4.1. Total Yield, Fresh and Dry Weight of White Head Cabbage and Outer Leaves

Cabbage is one of the most commonly produced vegetables globally, followed by carrots, onions, and beetroot [52]. Poland, after Romania (1.1 million tonnes), is the second-biggest producer of cabbage, accounting for 955 thousand tonnes in 2019 [53]. The outer leaves, generally are not used in food production and, together with the core of cabbage, are treated as waste and used as a green fertiliser, animal feed, or dietary fibre powder. Recently, outer leaves have become more attractive due to the possibility of recovery of bioactive compounds for the preparation of food additives [54].

In the major cases, there was a negative relationship between the foliar application of botanical extracts on the total yield, fresh and dry weight of white head cabbage. This may be associated with the presence of high epicuticular wax on their surface as well as the weak penetration of this layer by the prepared formulations. On the other hand, the increases in examined parameters were observed after the second spraying with botanical extracts. It can be therefore concluded that in the case of plants with a long growing season, the bio-products should be applied more than three times to achieve higher yields.

The highest yield of cabbage heads was observed in the groups treated with Hp H UAE while for outer cabbage leaves with Sg L UAE and Hp H UAE. On the other hand, the highest dry weight of cabbage heads was obtained after application of Hp H UAE and Vo R UAE. It can be seen from Table 1 that the use of Hp H HAE and Vo R UAE caused the lowest dry weight content of samples collected after the second spraying and the highest after the harvest. The opposite trend could be observed in the groups treated with Ur L UAE and To F MH. 

The treatment with Hp H UAE enhanced the antioxidant activity (assessed via ABTS assay) and vitamin C content in outer leaves and decreased in heads (compared with the control group), but at the same time, it decreased nitrates content in outer leaves and increased in heads. The use of Vo R UAE increased the antioxidant activity (assessed via DPPH and ABTS assays) in outer leaves and decreased in heads, while Ur L MH enhanced the antioxidant activity (assessed via ABTS assay) in leaves and lowered in heads. The application of To F MH increased the vitamin C content and antioxidant activity (assessed via ABTS assay) in leaves and lowered in heads, and decreased the antioxidant activity (assessed via FRAP assay) and nitrate content in leaves and increased them in heads.

These differences could result from the dose, concentration, and timing of applied extracts. Further research aimed at optimising the use of these innovative bio-products is needed to fully assess their effects on crops.

In our previous study, conducted on white head cabbage seedlings under laboratory conditions, it was shown that the highest yield of shoots was observed in the groups treated with Ur L UAE and To F UAE, while the highest root yields were seen with Vo R UAE, Sg L UAE, To F UAE, To L UAE, Hp H UAE, and Ur L UAE. On the other hand, the highest dry weight of shoots was obtained after application of To F UAE and Ur L UAE, while for roots it the highest values occurred after the application of Vo R UAE, Sg L UAE, and To L UAE [29,30]. The field trials on celeriac showed that the highest yield of leaves rosette was obtained after the application of Hp H MH, while for roots, the highest yield was after the application of Hp H MH. The highest dry weight of celeriac leaves rosette was noted in the group treated with Tp F MH and of roots with To F MH, To L MH, Tp F UAE, and Vo R MH [28].

In general, extracts based on *Hypericum perforatum*, *Solidago gigantea*, and *Taraxacum officinale* could be recommended as universal bio-products used to increase the total yield, whilst *Valeriana officinalis* and *Taraxacum officinale* to increase dry weight.

Our research confirmed the results of many works that the application of plant-based bio-products could increase the yield of crops in comparison to untreated control groups. For instance, the positive effects of (a) garlic extracts on the growth and weight of eggplant [55] and snap bean [56]; (b) moringa leaves extracts on cherry tomato [57], coriander [58], wheat [59], peas [60], and rocket [61]; (c) liquorice roots extract applied to common beans [62], onions [63], almonds [64], and fennel [65]; (d) red grape skin, blueberry fruits, and hawthorn leaf extracts on maize [66]; (e) lantana extracts on green gram [67].

Botanical bio-products can enhance plant growth and development by stimulating multiple physiological processes [68,69]. They constitute a rich source of biologically active compounds easily assimilable by crops [70,71]. The effects of their usage vary depending on the dose, concentration, application time and methods as well as crop species, development stage, and growth conditions [66,70].

### 4.2. Vitamin C

Vitamin C is a major water-soluble antioxidant essential for almost all living organisms. In humans, it protects the body from scurvy and diminishes the chance for diseases such as arteriosclerosis, cardiovascular diseases, and certain types of cancer. It is necessary to maintain good skin, gums and blood vessels, to form collagen, repair scar tissue, inhibit nitrosamine formation, absorb inorganic iron, reduce plasma cholesterol levels, and enhances the immune system [72,73,74]. Humans, along with other higher primates, guinea pigs, bats, several species of birds, insects, fish, and invertebrates, are incapable of synthesizing vitamin C [74,75,76]. The best source of vitamin C are plants that provide greater bioavailability of this vitamin than synthetic vitamin C (present in drugs or supplements) due to the content of various micronutrients and phytochemicals that can affect its assimilability [76]. For this reason, it is essential to obtain plants with enhanced vitamin C content. According to the US Department of Agriculture Agricultural Research Service (USDA), cabbage contains 36.6 mg·100 g^−1^ of vitamin C. As reported by the Food and Nutrition Board, the Recommended Dietary Allowance (RDA) for vitamin C is 75 mg·day^−1^ for women and 90 mg·day^−1^ for men [77,78]. In turn, in plants, vitamin C also plays a crucial role in, among other things, signal transduction and synthesis of the plant hormone ethylene; it controls the cell growth, elongation, division and programmed cell death and proper process of photosynthesis [75,76]. It can also enhance the resilience of crops to diverse stresses and extend postharvest shelf life [76].

The content of vitamin C was the most elevated in cabbage heads in the groups treated with To L UAE. It was observed that the amount of vitamin C was higher after the second spraying and lower after the harvest in comparison to the control group sprayed with water. This may be related to the nitrate content, which was lower in the outer leaves and higher in cabbage heads [79]. A similar trend could be observed in the case of the antioxidant activity measured using an ABTS assay.

The content of vitamin C in celeriac leaves rosette was the most elevated in groups treated with Hp H MH, while in roots, vitamin C was most elevated with Ur L UAE [28].

It could be seen that, depending on the vegetable species, obtained botanical extracts exerted diverse effects, and more research is required to select the best raw materials for the extraction to produce biostimulant of plant growth.

Other authors stated that the application of moringa extracts could enhance the ascorbic acid content in plum trees [80] and rocket [61], while the use of apple seeds, colza seeds, and rice husk extracts could increase this compound in kiwifruit [81].

The assumed mechanisms responsible for the increase in ascorbic acid content could be due to (1) the regulation of essential enzymes associated with the antioxidant homeostasis in cells and (2) the involvement in increased assimilation of macronutrients and micronutrients, which could redound to the synthesis of amino acids, tyrosine, and phenylalanine [82].

### 4.3. Total Phenolic Compounds

The human dietary intake of polyphenols at the level of 1 g per day was estimated and recommended by Kühnau in 1976, but despite the plethora of published works on their content in plants and intake evaluations [83], it remains hard to assess the proper reference intake [84]. The main source of polyphenols in the human diet is plant-based food such as vegetables, fruits, berries, cocoa, tea, coffee, and wine [83,84,85,86,87,88]. 

They constitute the biggest group of phytochemicals, and over 8000 phenolic compounds occur in the plant kingdom [8]. This group of compounds includes over 500 various molecules and, depending on the chemical structure, can be divided into four main classes: flavonoids, phenolic acids, stilbenes, and lignans [8,74,83,86,87,89]. Polyphenols exhibit numerous positive impacts on human health, especially antioxidant, anti-inflammatory, anticarcinogenic, antimicrobial, antiviral, antiallergic, antiproliferative, hepatoprotective, pro-apoptotic activity, and hormonal regulation capacity and have beneficial effects in type II diabetes, and cardiovascular and neurodegenerative diseases [1,13,74,84,86,87,90,91]. The absorption, bioavailability, and beneficial effects depend on the chemical structure of polyphenols [87,88]. The coloured phenolic compounds, such as anthocyanins, betalains, carotenoids, leucoanthocyanidin, and lycopenes, are strong antioxidants and may exhibit pharmacological properties [13,90]. Cabbage is considered a good source of phenolic compounds, such as flavonoids (mostly flavonols) and hydroxycinnamic acids [90].

It was shown that the total phenolic compounds content was stimulated to the greatest extent in cabbage heads with Vo R UAE and Sg L UAE.

The TPC content in cabbage seedlings shoots was the highest in groups treated with Ur L UAE [29,30], while in celeriac leaves rosettes, the highest TPC content was seen with Tp F MH, and in celeriac roots, the highest TPC content was seen with Hp H UAE [28]. As in the case of vitamin C, further studies should be considered to select appropriate raw materials.

The literature shows that the application of garlic-based extracts can increase the content of phenolics in faba beans [92]. The use of moringa-based extracts can also enhance the content of total phenolic content in coriander [58], anthocyanins in plum trees [80], and phenols in rocket [61]. The extracts based on red grape skin, blueberry fruits, and hawthorn leaves can increase the amount of phenolic acids in maize [66]. Mugwort extracts induced changes in the concentration of polyphenols in potatoes [93]. Borage leaves and flowers bio-products enhanced the total flavonoids and phenols content in lettuce [94]. French oak chips could increase the content of polyphenols in grapevines [95].

The effects of usage of biostimulants can be attributed to the induction of the activity of phenylalanine ammonia-lyase enzyme, which is an essential regulator in the phenols synthesis pathway [27]. Natural extracts can enhance the anthocyanin and phenolic content in crops as a result of the modulation of genes involved in the anthocyanin and flavonoid biosynthesis pathway [96]. The higher phenolic concentration and antioxidant capacity can be attributed as well to the presence of thiamine which evokes diverse genes that belong to the phenylpropanoid pathway with a resultant greater enhancement of secondary metabolites and antioxidant capacity [97]. The lower phenolic content may be associated with high nitrogen fertilisation [98].

### 4.4. The Antioxidant Activity (DPPH, ABTS, and FRAP)

Antioxidants are responsible for the delay of the oxidation of other molecules by inhibiting the initiation or propagation of oxidizing chain reactions by free radicals. They are also able to diminish the oxidative damage to the human body and thus reduce the risk of the development of various chronic diseases. It is reported that vegetables (e.g., beets, broccoli, cabbage, carrots, and kale) have high antioxidant activities, which may be attributed to the presence of nutrient antioxidants. The antioxidant activity of vegetable extracts is influenced by many factors, for example, the type and polarity of solvents, the isolation procedures, purity of active compounds, the assay techniques and type of substrate [99].

The increment in antioxidant activity measured using a DPPH assay in cabbage heads was noted in groups treated with Vo R MH, Sg L UAE, and Hp H UAE. Whilst using an ABTS assay, the highest antioxidant activity was obtained in cabbage heads in groups treated with Hp H MH. While using FRAP test, the highest activity was observed for cabbage heads in groups treated with Tp F UAE and Vo R MH.

The antioxidant activity in cabbage seedlings shoots measured using a DPPH assay was the highest in groups treated with Ur L UAE; using an ABTS assay after the application of To F UAE and Ur L UAE; while antioxidant activity was the highest using a FRAP assay after spraying with Ur L UAE [29,30]. In tests conducted on celeriac, the activity was the largest in leaves rosette after the use of Sg L MH (DPPH assay), Tp F UAE (ABTS assay), and Sg L MH (FRAP test). In the case of roots, the highest growth was noted in groups treated with Tp F UAE (DPPH test), To L UAE (ABTS test), and To F MH (FRAP test) [28].

The results demonstrated that *Urtica dioica*, *Taraxacum officinale*, and *Trifolium pratense* were the most commonly appearing raw materials responsible for the higher antioxidant activity of examined model plants.

Researchers stated that the application of moringa leaves extract can increase the radical scavenging activity in coriander [58], the antioxidant activity of plum trees [80], and the activity of antioxidants in quinoa [100]. The bio-products of alfalfa decreased the activity of antioxidant enzymes in maize [101].

Elevated antioxidant activities are essential for extending the shelf life and enhancing the nutritional quality of fresh food [97]. Phenolic compounds (e.g., carotenoids, anthocyanins, and flavonoids) have important health-related features and exhibit anticancer and antioxidant activities [102,103]. The increase in the antioxidant activity measured using a FRAP assay can be assigned to the activation of crucial enzymes connected with the homeostasis of cellular antioxidants and the improved absorption of the elements participating in the synthesis of amino acids [104].

### 4.5. Nitrates

The higher absorption of nitrates could be affected by a better-developed root system [70]. The nitrate content is one of the main factors of the quality of vegetables [105], and it can differ depending on the plant biological properties, the intensity of light, soil type, source of nitrogen temperature, humidity, population density, plant maturity, harvesting time, and storage time [106]. The main sources of nitrates in the human diet are raw vegetables (80%), with a smaller contribution from water (15%), animal products, and grains (5%). The impact of nitrate on human health is associated with their intake [106,107]. The reference daily intake set by the European Union is 3.7 mg·kg^−1^ of body weight per day, while the fatal adult dose is considered higher than 7–35 g [108]. Nitrates are relatively non–toxic; however, as a result of the activity of anaerobic bacteria in the oral cavity and gastrointestinal tract, 5%–20% of the ingested nitrate is transformed to toxic nitrite [105,106], which may lead to methemoglobinemia and carcinogenic nitrosamines [105]. The beneficial effect on the organism is associated with the conversion to NO, which improves cardiovascular health and supports gastrointestinal and immune functions [106].

The highest nitrate content in cabbage heads was noticed in groups treated with Tp F MH, To F UAE, Sg L MH, To L MH, and To L UAE. 

The botanical extracts application could result in the reduction in nitrate content in outer leaves due to the possible presence of specific bioactive compounds (e.g., amino acids and phytohormones). Bio-products could affect the activation of certain physiological processes in plants, as a result of which, in the initial stage of development, plants did not take up so many nutrients as those from the control group. A similar observation could be made in groups treated with a commercial biostimulant. The higher accumulation of nitrates in biostimulant-treated cabbage heads could be due to a more developed root system (biomass and root branching), which may have increased nitrate uptake and translocation. The use of botanical extracts could be a promising strategy that would benefit the environment by limiting the use of nitrogen fertilisers and livestock manure which is of particular importance in Poland due to the implementation of the Nitrates Directive.

With reference to literature data, in the case of celeriac, the biggest amount of examined parameter was observed after application of To L MH (for leaves rosette), and Tp F UAE and Ur L UAE (for roots) [28]. A comparison with data presented in this study revealed that *Trifolium pratense* and *Taraxacum officinale* affected the increase in nitrate content in model plants. In the case of other botanical extracts, borage extracts had no significant impact on the nitrate levels in lettuce [94].

The higher content of nitrates can be related to the decreased content of vitamin C in the samples [79,109]. A similar trend was observed in the case of the antioxidant activity measured via ABTS assay.

### 4.6. Macroelements, Microelements and, Toxic Elements

Vegetables are good sources of minerals which are crucial components for the proper metabolic activities of human body tissues, bones, teeth, blood, muscles, hair, and nerve cells. They are also required for the correct assimilation of vitamins [74,110]. Macroelements, required in larger amounts, especially vital for the body, are calcium, magnesium, potassium, sodium, iron, phosphorus, and chloride. Microelements needed in smaller quantities (less than 0.01% of the bodyweight), such as chromium, cobalt, copper, manganese, and zinc, are present in the environment at very low levels. The high amount of these metals might be toxic to the human body [74,111].

The application of botanical extracts resulted in the greatest content of macroelements in cabbage heads in the groups treated with To L MH and Sg L MH. In the case of microelements, the highest enrichment was noted in cabbage heads in groups treated with Sg L UAE and To L MH.

As compared with the literature data, the use of the following bio-products resulted in the greatest stimulation response in celeriac leaves rosette in the case of macroelements in the groups treated with Vo R UAE, Vo R MH, and Ur L MH; while in roots, the greatest response was seen with the application of Hp H MH, Tp F MH, Sg L UAE, and Ur L UAE. In the case of microelements, the highest elemental enrichment was noted in celeriac leaves rosette in the groups treated with Vo R UAE and Vo R MH; while the greatest enrichment in roots was with the application of To L UAE [28].

The results confirm that *Valeriana officinalis*, *Urtica dioica*, *Solidago gigantea*, and *Taraxacum officinale* could be a good choice for macroelement content enhancement, while *Valeriana officinalis*, *Solidago gigantea*, and *Taraxacum officinale* could be a good choice for microelement enhancement. In the available literature, it was found that the extracts based on moringa can increase the content of N, P, K, Ca, Mg, and Fe in rocket [61]. The increase in N, Mn, Fe, and Zn was also observed after the application of liquorice root extract in the cultivation of almonds [64]. The maize grains-based extracts can elevate the uptake of N, P, K, and Mg in sunflower seeds [112]. 

Biostimulants can improve the nutritional value of crops by affecting the availability of soil nutrients, soil properties (e.g., soil structure), plant nutrients uptake, their assimilation and translocation, as well as plant’s physiology (root morphology, root activity of H^+^ATPase, and root colonisation by arbuscular mycorrhizal fungi) [113,114,115,116].

### 4.7. Volatile Compounds

Essential oils are powerful compounds from natural sources, ordinarily plants, which are valued for their healing properties and prevention and treatment of cancer and cardiovascular diseases as well as antioxidant, antidiabetic, antiviral and antibacterial activities [117,118,119,120]. Moreover, essential oils and volatiles are natural, biodegradable, and display low toxicity to mammals [117]. It is estimated that over 3000 oils exhibit industrial significance [120] and are widely used as perfumes, flavours for food and beverages, pharmaceuticals, and cosmetics [117,118]. Ethereal oils are odorous and volatile chemical compounds present in 10% of plants in very low quantities (usually below 1%) are accumulated in special brittle secretory structures (e.g., glands, secretory hairs and cavities, secretory ducts, and resin ducts) [118]. Essential oils can be extracted from various plant parts (e.g., leaves, peels, barks, flowers, buds, and seeds) [119]. These oils play a crucial role in attracting opponents of herbivores to ensure the prevention of pathogens, attracting pollinators and disseminators to ease plant reproduction, plant-to-plant signalling, plant thermotolerance, etc. [120,121]. Simultaneously, they are not accumulating in the environment and possess a vast array of activities that reduce the risk of developing resistant pathogenic strains [117]. Volatile compounds (mainly mono-, sesqui-, and di-terpenes) constitute one of the most worthwhile plant compounds along with alkaloids and phenolic substances [117,122]. Factors like type and amount of constituents, abiotic factors, mineral nutrients, drought, light intensity, temperature, ozone, humidity, CO_2_ and density of planting affect the emission, odour, and flavour of oils [117,119,120,121].

The composition of volatile compounds was also altered after the use of the tested bio-products, for instance, in cabbage head groups treated with Sg L UAE, To L UAE, To F UAE, Vo R MH, Tp F UAE, and Hp H MH.

The application of botanical extracts modified the content of the volatile compounds (VCs) of celeriac leaves rosette as well (e.g., Sg L MH and Ur L UAE) [28]. It could be seen that botanical extracts differentially influenced the composition of individual plants. Bio-products that could be recommended for further investigation could be based on *Solidago gigantea* and *Taraxacum officinale* biomass. Extracts based on liquorice root were shown to improve the composition of essential oils in fennel [65], and the moringa extracts could increase the volatile oil yields of coriander [58].

### 4.8. Fatty Acids

In the majority of plants, the predominant unsaturated fatty acids (UFAs) are 18-carbon (C18) acids (oleate, linoleate, and α-linolenate [123,124]). These acids are components of membranes, modulators in glycerolipids and carbon and energy reserve in triacylglycerols, act as inherent antioxidants, precursors of diverse bioactive compounds and stocks of extracellular barrier constituents, and play a significant role in plant defence, biotic and abiotic stresses [124]. Furthermore, they show a variety of biological activities and physiological functions in the human body [124,125]. For instance, linoleate and α-linolenate acids are crucial because humans are unable to biosynthesise them. Additionally, their great potential for application in many branches of industry (e.g., in the production of biofuels, cosmetics, detergents, and pharmaceuticals) emphasises the importance of manipulating FA composition in crops [124]. On the other hand, polyunsaturated fatty acids (PUFAs) (omega-3 and omega-6 fatty acids) play a key role as healthy dietary bioactive compounds. A balanced consumption of PUFA may affect different aspects of immunity and metabolism [10].

The modification of fatty acids composition was observed in cabbage heads in groups treated with Sg L UAE, To F MH, To L MH, and Vo R UAE. In the case of celeriac roots, the use of extracts such as Hp H MH, Tp F MH, and To F UAE exerted the highest impact on the fatty acids composition [28]. Based on the obtained results, it was difficult to choose universal raw materials that would positively affect all desired parameters, and additional research is recommended. In the literature, it was noted that the maize grains bio-products increased sunflower seeds oil content (oleic and linoleic fatty acids) and decreased other saturated, mono-unsaturated, and polyunsaturated fatty acids [112].

### 4.9. Sterols

Phytosterols are similar in structure and function to cholesterol. These compounds are involved in the formation of cell membranes and are present in lower amounts in vegetables, fruits, cereals, nuts, etc. Generally, they occur in fat-rich or fat-soluble fractions of plant parts [8]. Among the dietary phytosterols, the most abundant are sitosterol (50%) and campesterol (33%). They exert numerous beneficial effects on disease prevention, e.g., certain types of cancer such as colon, breast, and prostate cancers. Sterols are also able to lower cholesterol levels [8,126].

The foliar spray with extracts changed the sterols composition of cabbage heads, which was most noticeable after the application of *Valeriana officinalis*.

### 4.10. Glucosinolates

Cruciferous vegetables contain more than 100 sulfur-containing glycosides, called jointly glucosinolates, which consist of a large group of secondary metabolites. The hydrolysis of glucosinolates by endogenous myrosinase can yield a variety of active products, such as epithionitriles, epithioalkanes, isothiocyanates, nitriles, oxazolidine-2-thiones, and thiocyanates [1,90,127,128,129,130,131]. The reaction depends on many factors, like the availability of ferrous ions, substrate, pH conditions, and the level and activity of specific protein factors [128]. The main groups of autolytic breakdown products are isothiocyanates and indoles, which have anti-carcinogenic activities [128,132,133]. Glucosinolates, due to their potent odour and taste, are involved in herbivores, and microbial defence systems whilst their breakdown products exert allelopathic, bactericidal, fungicidal, and nematocidal activities [127,133,134]. Early-stage research was mostly concerned with the negative impact on the human body (embryonic death, slowing of growth, inhibition of thyroid activity, low foetal weight, and damage to the thyroid, liver, kidneys, and pancreas), but further studies stated that lower intake might exhibit important health-promoting effects like anticancer (colon, oesophagus, lung, breast, and uterine) and antioxidant as well as a beneficial impact on gut microflora [52,127,129,130,134]. The total glucosinolates content in a white head cabbage amounts to 1.05–70.56 µmol·g^-1^ of dry weight [90]. 

Our finding proved that the application of botanical extracts did not stimulate the composition of glucosinolates.

### 4.11. Sugars

Cabbage can be consumed in several forms (fresh, fermented, baked, etc.), whilst sauerkraut is especially highly valued in Poland, Germany, and Eastern Europe. According to the statistics, the average Pole consumes 11 kg of raw cabbage and 6 kg of sauerkraut per year [52]. Fermentation is one of the techniques to preserve food and retain the nutritional quality of vegetables during the offseason [135]. The sauerkraut fermentation process is influenced by cabbage surface microflora and its sugar content [136]. Generally, cabbage contains 4–5% sugar (2.5% glucose and 2% fructose) which during fermentation diffuses in brine and its concentration rises [135].

Our results suggest that *Taraxacum officinale* (flowers) and *Hypericum perforatum* could be used for increasing the sugar content.

Similar results were also found for other plant-based extracts; for example, the moringa extracts could increase the cherry tomato fruit concentration of soluble sugars [57], total sugars in coriander [58] and rocket [61]. The red grape skin, blueberry fruits, and hawthorn leaf extracts could increase the sugars content in maize [66], while maize grains extracts could improve the total soluble sugars in sunflower seeds [112]. Borage extracts showed a lack of significant impact on the level of sugars [94].

### 4.12. The Explanation of the Differential Impact of the Botanical Extracts

Biostimulants contain a vast array of bioactive compounds (hormones, peptides, phenolic compounds, saccharides etc.) affecting the diverse physiological processes responsible for the stimulation of plant growth, the increment of nutrient use efficiency, resistance to abiotic stress and to biotic stresses, and the reduction in the use of chemical fertilisers. The application of these types of products does not cause an unfavourable impact on crop yield or quality [24,137,138]. In general, the hypothesised mechanisms of their action could be related to the modulation of gene expression, stimulation of amino acid biosynthesis, as well as enhancing antioxidant, osmolyte, protein, or pigment content [137]. To identify their mode of action, data concerning the detailed morphological, physiological, biochemical, and molecular analyses are required. However, it might be challenging because they are derived mainly from complex sources containing multiple bioactive compounds that, together, may contribute to specific effects in plants [139]. The difference between the biostimulants effects can be attributable to many factors, e.g., extracts per se, plant genotype, weather conditions, cultivation methods, harvesting time, maturity etc. [138]. Despite the recent advancements in deciphering their physiological and biochemical mechanisms, additional studies are still necessary to understand, for instance, which molecular mechanisms underlie the observed biostimulatory action, or what is the optimal method, dose, and time of their application for enhancing plant growth, development and resilience to stresses. Taking into account that there seem to be more questions than answers, the findings attempting to unravel the complex mechanisms should combine the interaction between the scientific community and the private industry. This will allow the development of second-generation specific plant biostimulants [140]. 

The application of plant biostimulants appears to be the best method to meet the urgent need in organic agricultural methods based on bioactive, eco-friendly, and safe substances [24]. Therefore, research on new potential biostimulants should last several years to be able to select the most promising raw materials for their production. Nonetheless, we believe that the examination of the potential of higher plants for the production of innovative biostimulants is fully justified. Our study has shed light on the production and application of innovative plant extracts that could increase quality attributes of crops and, as a consequence, provide higher incomes for producers and safe and nourishing food for consumers.

## 5. Conclusions

Plant biostimulants could make a significant contribution to ecologically and economically sustainable crop production systems as a novel and potential category of agricultural inputs. This approach can complement the range of products available on the market, mainly synthetic fertilisers, and fortify crops with essential nutrients and bioactive compounds, which may be beneficial in health-related problems. Thus, there is a great potential for the use of higher plants, commonly occurring in nature, for the development of potential biostimulants. In addition, the improvement in extraction techniques will be crucial towards a sustainable method for their production.

Extensive research that has been carried out shows that higher plants are a good source of bioactive compounds and can be used in the production of potential biostimulants. Obtained formulations exhibited a significant impact on the growth and physiological parameters of white head cabbage. Generally, the foliar application of botanical extracts did not have a statistically significant effect on the yield of heads (excluding St. John’s wort) or their dry weight (excluding St. John’s wort and valerian) but did on the yield of outer leaves (e.g., giant goldenrod and St. John’s wort) and their dry weight (e.g., flowers of common dandelion and nettle). An increase was observed in the content of photosynthetic pigments (e.g., leaves and flowers of common dandelion, St. John’s wort, and giant goldenrod). The content of vitamin C was enhanced in samples after a second spraying (e.g., red clover, giant goldenrod, and the flowers of common dandelion) but diminished in heads after a third spraying (excluding leaves of common dandelion). In the majority of cases, the tested extracts did not enhance the amount of total phenolic compounds (excluding valerian and giant goldenrod). The antioxidant activity measured was using DPPH, ABTS, and FRAP assays demonstrated that bio-products had a differential impact on plants. The tested botanical extracts tend to diminish the content of nitrates in samples after the second spraying (e.g., giant goldenrod, common dandelion, and valerian) but increased in cabbage heads (e.g., red clover, flowers, and the leaves of common dandelion and giant goldenrod). The enrichment with macroelements and microelements in heads was observed. The foliar application of the extracts exerted a varied impact on the composition of volatile compounds, fatty acids, and sterols. Bio-products raised the content of reducing sugars (e.g., flowers of common dandelion and St. John’s wort) but had a smaller impact on the content of total sugars (e.g., St. John’s wort and flowers of common dandelion).

On the basis of the promising findings presented in this paper, work on their impact on different plants and under different environmental conditions are recommended.

## Figures and Tables

**Table 1:** Effect of the foliar application of the botanical extracts on the average fresh weight of cabbage head and outer leaves after harvest, the total yield of cabbage (*n* = 3 *, mean ± SD), and dry weight of leaves after second spraying and cabbage head after harvest (*n* = 3, mean ± SD).

Group	Fresh Weight, Head < 1.2 kg		Fresh Weight, Head > 1.2 kg		Total Yield		Total Yield Minus Non-Marketable Yield	Dry Weight	
	Head	Outer Leaves	Head	Outer Leaves	Head	Outer Leaves		After Second Spraying	After Harvest
	kg		kg		t·ha^−1^		%	%	
C	0.66 ± 0.12 c	0.88 ± 0.15	2.61 ± 0.21	1.51 ± 0.17	65.28 ± 8.50	39.61 ± 5.57	100	12.28 ± 0.21 b	10.08 ± 0.25
CF	0.73 ± 0.12 c	0.75 ± 0.11	2.53 ± 0.47	1.50 ± 0.11	64.26 ± 11.47 c	38.99 ± 4.25 c	100	12.99 ± 0.38 a	9.53 ± 0.19
CB	0.48 ± 0.06 a,b	0.81 ± 0.16	2.74 ± 0.37	1.60 ± 0.14	71.85 ± 9.49 b	43.08 ± 6.00 b	100	12.55 ± 0.20	9.76 ± 0.34
Hp H UAE	0.83 ± 0.09 a,c	0.93 ± 0.18 b	2.84 ± 0.16 b	1.63 ± 0.17	73.97 ± 6.17 a,b	43.66 ± 6.41 a,b	100	11.35 ± 0.33 a,b,c	10.45 ± 0.19 b,c
Hp H MH	0.90 ± 0.14 a,b,c	1.09 ± 0.19 a,b,c	2.39 ± 0.06 c	1.52 ± 0.13	60.59 ± 5.52 c	40.64 ± 5.13	100	12.74 ± 0.21	9.60 ± 0.34
Sg L UAE	0.79 ± 0.08 a,c	0.76 ± 0.12	2.50 ± 0.38	1.78 ± 0.30 a,b,c	63.73 ± 8.14 c	46.11 ± 7.62 a,b	100	12.36 ± 0.22 b	9.71 ± 0.18
Sg L MH	0.50 ± 0.08 a,b	0.80 ± 0.15	2.23 ± 0.09 a,b,c	1.65 ± 0.22 b	56.31 ± 5.86 a,b,c	43.05 ± 6.85 b	100	12.55 ± 0.33	10.07 ± 0.37
To F UAE	0.76 ± 0.18 c	0.99 ± 0.18 b,c	2.31 ± 0.43 a,c	1.38 ± 0.10 c	57.19 ± 12.16 a,b,c	36.65 ± 4.58 c	100	12.94 ± 0.21 a	9.74 ± 0.33
To F MH	0.63 ± 0.08 c	0.67 ± 0.09 a,c	2.65 ± 0.27	1.53 ± 0.10	69.70 ± 8.27	40.82 ± 4.29	100	13.53 ± 0.25 a,c	9.48 ± 0.19
To L UAE	0.90 ± 0.14 a,b,c	0.65 ± 0.10 a,c	2.49 ± 0.19	1.57 ± 0.09	64.79 ± 7.64 c	41.02 ± 4.43	100	12.65 ± 0.20	10.05 ± 0.44
To L MH	0.87 ± 0.08 a,b,c	0.85 ± 0.10	2.51 ± 0.28	1.61 ± 0.17	61.77 ± 6.30 c	40.96 ± 4.51	100	12.95 ± 0.29 a	9.99 ± 0.28
Tp F UAE	0.99 ± 0.18 a,b,c	0.99 ± 0.07 b,c	2.62 ± 0.19	1.54 ± 0.17	66.52 ± 8.45	40.64 ± 3.59	100	12.68 ± 0.25	9.69 ± 0.24
Tp F MH	0.61 ± 0.14 b,c	0.81 ± 0.14	2.60 ± 0.26	1.67 ± 0.15 a,b	64.80 ± 10.63 c	43.10 ± 5.47 b	100	12.64 ± 0.21	9.66 ± 0.38
Ur L UAE	0.88 ± 0.12 a,b,c	1.19 ± 0.20 a,b,c	2.35 ± 0.47 c	1.31 ± 0.14 a,b,c	61.17 ± 10.37 c	36.05 ± 4.94 c	100	13.20 ± 0.24 a,c	10.03 ± 0.23
Ur L MH	0.81 ± 0.19 a,c	0.71 ± 0.14 a	2.67 ± 0.49	1.59 ± 0.16	67.21 ± 13.85	40.88 ± 6.07	100	12.86 ± 0.19	9.10 ± 0.31 a,c
Vo R UAE	0.91 ± 0.18 a,b,c	1.21 ± 0.23 a,b,c	2.45 ± 0.38	1.56 ± 0.07	61.46 ± 10.98 c	41.95 ± 4.88	100	12.20 ± 0.37 b	10.51 ± 0.20 b,c
Vo R MH	0.44 ± 0.07 a,b	0.77 ± 0.07	2.44 ± 0.37 c	1.45 ± 0.03 c	64.18 ± 10.24 c	38.97 ± 2.18 c	100	12.89 ± 0.20 a	9.89 ± 0.23

Statistically significant differences (*p* < 0.05) (a) between the control group (C) and the botanical extracts; (b) between the formulation (CF) and the botanical extracts; (c) between commercial biostimulant (CB) and the botanical extracts; Hp H, *Hypericum perforatum* L. (St. John’s wort, herb); UAE, ultrasound-assisted extraction; MH, mechanical homogenisation; Sg L, *Solidago gigantea* Ait. (giant goldenrod, leaf); To F, To L, *Taraxacum officinale* (L.) Weber ex F.H. Wigg (common dandelion, flower, leaf); Tp F, *Trifolium pratense* L. (red clover, flower); Ur L, *Urtica dioica* L. (nettle, leaf); Vo R, *Valeriana officinalis* L. (valerian, root). * Three replications (plots) in each experimental group and each consisted of 20 plants.

**Table 2:** Effect of the foliar application of the botanical extracts on the chlorophyll *a* + *b* and carotenoids content in leaves after second spraying (*n* = 4, mean ± SD), the *L*, *a*, *b* and SPAD values of leaves after second spraying (*n* = 16, mean ± SD), and vitamin C content in leaves after second spraying and cabbage head after harvest (*n* = 4, mean ± SD).

Group	Chlorophyll *a* + *b*	Carotenoids	Leaf Colour			Greenness Index of the Leaves	Vitamin C	
	mg·g^−1^ FW	µg·g^−1^ FW	*L*	*a*	*b*	SPAD Value	mg·100g^−1^ FW	
	After Second Spraying							After Harvest
C	1.24 ± 0.05	30.77 ± 1.62	40.75 ± 1.64 b,c	−6.29 ± 0.51 c	10.91 ± 1.69 c	65.96 ± 3.80 c	97.40 ± 3.31 c	54.80 ± 3.46 c
CF	1.34 ± 0.03	27.74 ± 1.04	48.03 ± 1.85 a	−6.73 ± 0.50	11.58 ± 1.39	63.08 ± 3.08	103.43 ± 4.26 c	53.70 ± 2.15 c
CB	1.46 ± 0.04	31.44 ± 1.30	46.66 ± 1.61 a	−7.19 ± 0.58 a	13.59 ± 1.89 a	60.89 ± 3.26 a	117.73 ± 4.28 a,b	41.40 ± 2.84 a,b
Hp H UAE	1.42 ± 0.04	32.61 ± 1.59	42.71 ± 2.58 b,c	−6.93 ± 0.45	12.63 ± 1.85	68.65 ± 2.78 b,c	113.53 ± 5.35 a	46.07 ± 2.66 a,b
Hp H MH	1.62 ± 0.06 a,b	32.83 ± 1.03	43.07 ± 2.38 b,c	−6.21 ± 0.46 c	9.97 ± 1.16 c	69.81 ± 3.47 b,c	112.43 ± 5.99 a	51.13 ± 2.82 c
Sg L UAE	1.56 ± 0.07 a,b	33.59 ± 1.72 b	42.27 ± 1.93 b,c	−6.03 ± 0.20 b,c	9.42 ± 1.10 b,c	65.51 ± 2.71 c	97.07 ± 4.49 c	52.83 ± 3.64 c
Sg L MH	1.55 ± 0.08 a	34.03 ± 1.41 b	45.61 ± 1.97 a	−6.71 ± 0.37	11.94 ± 1.31	72.59 ± 3.59 a,b,c	127.60 ± 4.70 a,b	47.67 ± 3.76 a
To F UAE	1.32 ± 0.03	30.05 ± 1.62	43.46 ± 1.69 b,c	−6.81 ± 0.62	12.92 ± 1.36	66.25 ± 2.35 c	126.60 ± 6.65 a,b	45.47 ± 1.29 a,b
To F MH	1.63 ± 0.04 a,b	33.70 ± 1.17 b	46.95 ± 1.20 a	−6.15 ± 0.38 c	9.67 ± 1.34 c	74.20 ± 3.90 a,b,c	125.87 ± 3.82 a,b	43.47 ± 2.86 a,b
To L UAE	1.59 ± 0.09 a,b	34.93 ± 1.84 b	39.44 ± 2.50 b,c	−6.13 ± 0.43 c	10.65 ± 1.20 c	70.93 ± 4.79 a,b,c	106.57 ± 5.15	69.93 ± 4.33 a,b,c
To L MH	1.81 ± 0.09 a,b,c	37.47 ± 1.41 a,b,c	43.82 ± 1.75 a,b,c	−6.28 ± 0.50 c	10.64 ± 1.21 c	67.65 ± 3.37 b,c	118.03 ± 5.93 a,b	54.53 ± 2.50 c
Tp F UAE	1.48 ± 0.07 a	33.94 ± 1.62 b	43.32 ± 2.25 b,c	−6.39 ± 0.54 c	10.36 ± 1.08 c	68.56 ± 2.51 b,c	104.93 ± 6.97 c	50.27 ± 3.35 c
Tp F MH	1.46 ± 0.04	30.03 ± 1.47	45.64 ± 1.48 a	−7.05 ± 0.52 a	13.17 ± 1.71 a	67.05 ± 3.28 c	148.17 ± 7.67 a,b,c	51.23 ± 2.02 c
Ur L UAE	1.43 ± 0.07	30.95 ± 1.24	41.42 ± 1.93 b,c	−6.08 ± 0.35 c	10.11 ± 1.48 c	68.61 ± 3.83 b,c	100.20 ± 4.35 c	42.03 ± 2.65 a,b
Ur L MH	1.44 ± 0.05	30.01 ± 1.01	44.72 ± 1.76 a,b	−6.32 ± 0.48 c	10.37 ± 1.29 c	71.08 ± 4.64 a,b,c	115.23 ± 5.86 a	51.33 ± 2.77 c
Vo R UAE	1.52 ± 0.07 a	31.42 ± 1.39	41.85 ± 1.55 b,c	−6.34 ± 0.52 c	11.00 ± 1.69 c	68.56 ± 2.57 b,c	96.63 ± 5.84 c	54.67 ± 4.09 c
Vo R MH	1.36 ± 0.02	29.42 ± 1.52	42.25 ± 1.32 b,c	−6.19 ± 0.42 c	10.08 ± 1.41 c	71.03 ± 2.29 a,b,c	111.93 ± 4.74 a	53.20 ± 1.66 c

Statistically significant differences (*p* < 0.05) (a) between the control group (C) and the botanical extracts; (b) between the formulation (CF) and the botanical extracts; (c) between commercial biostimulant (CB) and the botanical extracts; Hp H, *Hypericum perforatum* L. (St. John’s wort, herb); Sg L, *Solidago gigantea* Ait. (giant goldenrod, leaf); To F, To L, *Taraxacum officinale* (L.) Weber ex F.H. Wigg (common dandelion, flower, leaf); Tp F, *Trifolium pratense* L. (red clover, flower); Ur L, *Urtica dioica* L. (nettle, leaf); Vo R, *Valeriana officinalis* L. (valerian, root). The *L* value in leaf colour indicates the level of light (numbers from 51 to 100) or dark (numbers from 0 to 50). The *a* value in leaf colour indicates redness (positive number) or greenness (negative number). The *b* value in leaf colour indicates yellowness (positive number) or blueness (negative number).

**Table 3:** Effect of the foliar application of the botanical extracts on the total phenolic compounds content and the antioxidant activity (DPPH assay, ABTS assay, and FRAP assay) of leaves after second spraying and cabbage head after harvest (*n* = 4, mean ± SD).

Group	Total Phenolic Compounds		DPPH Assay		ABTS Assay		FRAP Assay	
	mg GAE·100 g^−1^ FW		µM Trolox·g^−1^ FW					
	After Second Spraying	After Harvest	After Second Spraying	After Harvest	After Second Spraying	After Harvest	After Second Spraying	After Harvest
C	167.98 ± 8.47 b,c	82.95 ± 5.34 b,c	1.37 ± 0.10	0.45 ± 0.09 b,c	11.03 ± 0.64 b,c	3.90 ± 0.60	4.84 ± 0.27	1.23 ± 0.08 c
CF	126.26 ± 5.30 a,c	51.43 ± 2.56 a,c	1.23 ± 0.13	0.24 ± 0.06 a	14.93 ± 0.57 a	2.36 ± 0.35	4.67 ± 0.20	1.41 ± 0.07
CB	99.24 ± 7.60 a,b	69.65 ± 4.74 a,b	1.47 ± 0.11	0.14 ± 0.03 a	13.26 ± 0.66 a	2.61 ± 0.52	4.00 ± 0.17	1.53 ± 0.06 a
Hp H UAE	161.22 ± 6.35 b,c	57.71 ± 1.97 a,c	1.44 ± 0.13	0.52 ± 0.08 b,c	16.06 ± 0.52 a,c	1.82 ± 0.31 a	6.48 ± 0.35 a,b,c	1.39 ± 0.09
Hp H MH	108.87 ± 7.00 a	71.11 ± 4.39 a,b	1.20 ± 0.07	0.45 ± 0.06 b,c	16.38 ± 0.67 a,c	5.76 ± 0.47 a,b,c	4.14 ± 0.15	1.33 ± 0.04
Sg L UAE	178.30 ± 9.65 b,c	88.71 ± 4.47 b,c	1.51 ± 0.12	0.63 ± 0.07 b,c	14.18 ± 0.65 a	2.49 ± 0.56	6.16 ± 0.51 a,b,c	1.43 ± 0.07 a
Sg L MH	149.39 ± 5.87 a,b,c	83.44 ± 5.65 b,c	1.13 ± 0.07 c	0.39 ± 0.08 c	18.46 ± 0.42 a,b,c	1.96 ± 0.43 a	6.10 ± 0.32 a,b,c	1.46 ± 0.05 a
To F UAE	129.23 ± 10.70 a,c	73.81 ± 4.28 b	1.23 ± 0.08	0.33 ± 0.05 c	12.77 ± 0.76 b	1.09 ± 0.26 a	5.30 ± 0.16 c	1.52 ± 0.04 a
To F MH	103.83 ± 5.36 a,b	61.02 ± 4.02 a	1.34 ± 0.11	0.32 ± 0.06	14.79 ± 0.63 a	2.44 ± 0.52	4.30 ± 0.24	1.53 ± 0.09 a
To L UAE	139.07 ± 8.70 a,c	79.95 ± 3.38 b,c	1.66 ± 0.09	0.38 ± 0.08 c	13.74 ± 1.01 a	2.12 ± 0.30 a	4.89 ± 0.33	1.35 ± 0.08
To L MH	137.59 ± 9.79 a,c	66.88 ± 4.44 a,b	1.12 ± 0.10 c	0.36 ± 0.04 c	15.81 ± 0.65 a,c	2.71 ± 0.31	4.17 ± 0.41	0.91 ± 0.04 a,b,c
Tp F UAE	163.43 ± 5.78 b,c	84.21 ± 3.35 b,c	0.95 ± 0.11 a,c	0.28 ± 0.04	13.90 ± 0.87 a	2.21 ± 0.47 a	6.21 ± 0.39 a,b,c	1.69 ± 0.06 a,b
Tp F MH	123.70 ± 7.03 a,c	62.34 ± 4.77 a,b	0.91 ± 0.07 a,b,c	0.22 ± 0.05 a	15.37 ± 0.90 a,c	1.46 ± 0.26 a	4.48 ± 0.28	1.44 ± 0.07 a
Ur L UAE	137.00 ± 8.25 a,c	66.85 ± 3.82 a,b	0.94 ± 0.12 a,c	0.40 ± 0.06 c	17.27 ± 0.45 a,b,c	1.71 ± 0.45 a	5.74 ± 0.36 b,c	1.35 ± 0.10
Ur L MH	87.85 ± 6.32 a,b	58.30 ± 3.98 a,c	1.25 ± 0.09	0.33 ± 0.06	14.70 ± 0.59 a	2.53 ± 0.55	4.06 ± 0.23	1.31 ± 0.08 c
Vo R UAE	234.60 ± 8.24 a,b,c	111.68 ± 6.39 a,b,c	1.60 ± 0.07 b	0.19 ± 0.04 a	13.09 ± 0.60 a	1.93 ± 0.41 a	4.71 ± 0.31	1.14 ± 0.04 b,c
Vo R MH	142.42 ± 7.97 a,c	88.62 ± 3.55 b,c	1.41 ± 0.09	0.67 ± 0.07 a,b,c	14.74 ± 0.84 a	1.92 ± 0.27 a	5.71 ± 0.34 b,c	1.68 ± 0.07 a,b

Statistically significant differences (*p* < 0.05) (a) between the control group (C) and the botanical extracts; (b) between the formulation (CF) and the botanical extracts; (c) between commercial biostimulant (CB) and the botanical extracts; Hp H, *Hypericum perforatum* L. (St. John’s wort, herb); Sg L, *Solidago gigantea* Ait. (giant goldenrod, leaf); To F, To L, *Taraxacum officinale* (L.) Weber ex F.H. Wigg (common dandelion, flower, leaf); Tp F, *Trifolium pratense* L. (red clover, flower); Ur L, *Urtica dioica* L. (nettle, leaf); Vo R, *Valeriana officinalis* L. (valerian, root).

**Table 4:** Effect of the foliar application of the botanical extracts on the nitrates content in leaves after second spraying and cabbage head after harvest (*n* = 4, mean ± SD), and macroelements content of cabbage head after harvest (*n* = 3, mean ± SD).

Treatment	Nitrates		N	P	K	Ca	Mg	S
	mg·kg ^−1^ FW		g·kg^−1^ DW					
	After Second Spraying	After Harvest	After Harvest					
C	1326.18 ± 128.09 b,c	102.76 ± 11.19	23.83 ± 1.19	2.53 ± 0.13 b,c	24.37 ± 1.22	5.60 ± 0.28	0.95 ± 0.05	7.88 ± 0.39
CF	796.96 ± 98.21 a	122.59 ± 14.69	27.05 ± 1.35	2.06 ± 0.10 a	23.33 ± 1.17	6.20 ± 0.31	1.02 ± 0.05	8.58 ± 0.43
CB	748.99 ± 96.50 a	136.77 ± 13.99	26.70 ± 1.34	1.94 ± 0.10 a	23.94 ± 1.20	6.00 ± 0.30	1.02 ± 0.05	8.65 ± 0.43
Hp H UAE	833.50 ± 130.75 a	160.62 ± 16.83 a	24.15 ± 1.21	1.94 ± 0.10 a	23.13 ± 1.16	6.54 ± 0.33	1.03 ± 0.05	8.15 ± 0.41
Hp H MH	764.64 ± 77.98 a	162.73 ± 25.19 a	25.40 ± 1.27	1.66 ± 0.08 a,b	22.70 ± 1.13	6.31 ± 0.32	0.96 ± 0.05	8.74 ± 0.44
Sg L UAE	1104.25 ± 167.26 b,c	129.86 ± 14.76	26.75 ± 1.34	1.97 ± 0.10 a	24.11 ± 1.21	6.50 ± 0.32	1.01 ± 0.05	8.18 ± 0.41
Sg L MH	557.47 ± 72.12 a	189.96 ± 25.23 a,b,c	23.03 ± 1.15 b	1.28 ± 0.06 a,b,c	20.80 ± 1.04 a	7.20 ± 0.36 a,c	1.07 ± 0.05	8.88 ± 0.44
To F UAE	725.91 ± 51.75 a	195.19 ± 21.52 a,b,c	25.80 ± 1.29	1.94 ± 0.10 a	22.17 ± 1.11	6.66 ± 0.33 a	0.99 ± 0.05	8.21 ± 0.41
To F MH	861.37 ± 127.27 a	169.08 ± 24.03 a,b	25.50 ± 1.28	1.56 ± 0.08 a,b,c	23.42 ± 1.17	6.57 ± 0.33	1.05 ± 0.05	8.33 ± 0.42
To L UAE	1321.81 ± 77.59 b,c	177.81 ± 16.23 a,b	22.78 ± 1.14 b,c	1.44 ± 0.07 a,b,c	22.97 ± 1.15	7.62 ± 0.38 a,b,c	1.06 ± 0.05	8.23 ± 0.41
To L MH	617.41 ± 100.63 a	179.93 ± 21.98 a,b,c	26.75 ± 1.34	1.81 ± 0.09 a	22.66 ± 1.13	7.12 ± 0.36 a,c	1.07 ± 0.05	8.76 ± 0.44
Tp F UAE	1052.38 ± 115.77 a,b,c	135.28 ± 18.19	24.23 ± 1.21	1.94 ± 0.10 a	22.48 ± 1.12	6.94 ± 0.35 a	1.03 ± 0.05	8.10 ± 0.41
Tp F MH	842.92 ± 116.24 a	293.86 ± 22.13 a,b,c	23.13 ± 1.16 b	2.00 ± 0.10 a	22.39 ± 1.12	7.62 ± 0.38 a,b,c	1.12 ± 0.06 a	8.30 ± 0.42
Ur L UAE	892.42 ± 62.39 a	137.64 ± 13.27	25.35 ± 1.27	1.94 ± 0.10 a	22.81 ± 1.14	6.95 ± 0.35 a	1.08 ± 0.05	8.66 ± 0.43
Ur L MH	1067.00 ± 146.53 a,b,c	125.33 ± 17.28	24.43 ± 1.22	2.00 ± 0.10 a	21.72 ± 1.09	5.87 ± 0.29	0.98 ± 0.05	8.05 ± 0.40
Vo R UAE	902.95 ± 90.50 a	105.25 ± 9.87	24.60 ± 1.23	1.78 ± 0.09 a	22.49 ± 1.12	5.98 ± 0.30	0.97 ± 0.05	7.94 ± 0.40
Vo R MH	663.62 ± 130.34 a	161.80 ± 14.07 a	24.28 ± 1.21	1.38 ± 0.07 a,b,c	23.03 ± 1.15	7.09 ± 0.35 a,c	1.03 ± 0.05	8.00 ± 0.40

Statistically significant differences (*p* < 0.05) (a) between the control group (C) and the botanical extracts; (b) between the formulation (CF) and the botanical extracts; (c) between commercial biostimulant (CB) and the botanical extracts; Hp H, *Hypericum perforatum* L. (St. John’s wort, herb); Sg L, *Solidago gigantea* Ait. (giant goldenrod, leaf); To F, To L, *Taraxacum officinale* (L.) Weber ex F.H. Wigg (common dandelion, flower, leaf); Tp F, *Trifolium pratense* L. (red clover, flower); Ur L, *Urtica dioica* L. (nettle, leaf); Vo R, *Valeriana officinalis* L. (valerian, root).

**Table 5:** Effect of the foliar application of the botanical extracts on the microelements and toxic elements content of cabbage head after harvest (*n* = 3, mean ± SD).

Treatment	Fe	Cu	Zn	Mn	Ni	Cd	Pb
	mg·kg^−1^ DW						
C	41.88 ± 2.09	3.25 ± 0.16	21.93 ± 1.10	18.75 ± 0.94 b	2.39 ± 0.12 b,c	0.09 ± 0.00	3.41 ± 0.17
CF	40.88 ± 2.04 c	3.31 ± 0.17	24.44 ± 1.22	16.00 ± 0.80 a,c	3.18 ± 0.16 a,c	0.08 ± 0.00	3.88 ± 0.19
CB	47.50 ± 2.38 b	3.53 ± 0.18	23.94 ± 1.20	18.75 ± 0.94 b	4.13 ± 0.21 a,b	0.09 ± 0.00	3.45 ± 0.17
Hp H UAE	40.50 ± 2.03 c	2.88 ± 0.14 c	19.80 ± 0.99 b,c	15.63 ± 0.78 a,c	2.86 ± 0.14 a,c	0.15 ± 0.01 a,b,c	4.38 ± 0.22 a,c
Hp H MH	38.00 ± 1.90 c	2.98 ± 0.15 c	22.09 ± 1.10	18.75 ± 0.94 b	2.71 ± 0.14 b,c	0.10 ± 0.01 b	3.49 ± 0.17
Sg L UAE	45.13 ± 2.26	3.20 ± 0.16	23.09 ± 1.15	18.13 ± 0.91	2.94 ± 0.15 a,c	0.11 ± 0.01 a,b,c	4.23 ± 0.21 a,c
Sg L MH	35.25 ± 1.76 a,c	2.71 ± 0.14 a,b,c	20.78 ± 1.04 b	16.88 ± 0.84	2.85 ± 0.14 a,c	0.15 ± 0.01 a,b,c	3.84 ± 0.19
To F UAE	39.63 ± 1.98 c	3.30 ± 0.17	21.24 ± 1.06	18.63 ± 0.93	2.93 ± 0.15 a,c	0.15 ± 0.01 a,b,c	4.08 ± 0.20 a,c
To F MH	41.88 ± 2.09	3.18 ± 0.16	22.13 ± 1.11	16.63 ± 0.83	3.43 ± 0.17 a,c	0.09 ± 0.00	3.74 ± 0.19
To L UAE	37.38 ± 1.87 c	2.83 ± 0.14 b,c	19.83 ± 0.99 b,c	16.50 ± 0.83	2.50 ± 0.13 b,c	0.15 ± 0.01 a,b,c	4.45 ± 0.22 a,c
To L MH	47.00 ± 2.35	3.06 ± 0.15	22.10 ± 1.11	18.63 ± 0.93	3.36 ± 0.17 a,c	0.14 ± 0.01 a,b,c	3.86 ± 0.19
Tp F UAE	33.50 ± 1.68 a,b,c	2.84 ± 0.14 b,c	19.09 ± 0.95 b,c	16.63 ± 0.83	2.49 ± 0.12 b,c	0.16 ± 0.01 a,b,c	3.76 ± 0.19
Tp F MH	34.13 ± 1.71 a,b,c	2.63 ± 0.13 a,b,c	21.45 ± 1.07	15.38 ± 0.77 a,c	2.76 ± 0.14 c	0.11 ± 0.01 a,b,c	3.57 ± 0.18
Ur L UAE	39.75 ± 1.99 c	3.01 ± 0.15 c	21.69 ± 1.08	17.00 ± 0.85	2.64 ± 0.13 b,c	0.13 ± 0.01 a,b,c	4.07 ± 0.20 a,c
Ur L MH	38.75 ± 1.94 c	3.05 ± 0.15 c	21.56 ± 1.08	17.13 ± 0.86	2.74 ± 0.14 c	0.10 ± 0.01 b	3.38 ± 0.17
Vo R UAE	39.00 ± 1.95 c	3.06 ± 0.15	22.09 ± 1.10	18.50 ± 0.93	2.39 ± 0.12 b,c	0.09 ± 0.00	3.71 ± 0.19
Vo R MH	47.88 ± 2.39 b	2.91 ± 0.15 c	21.14 ± 1.06	17.00 ± 0.85	2.53 ± 0.13 b,c	0.14 ± 0.01 a,b,c	3.94 ± 0.20

Statistically significant differences (*p* < 0.05) (a) between the control group (C) and the botanical extracts; (b) between the formulation (CF) and the botanical extracts; (c) between commercial biostimulant (CB) and the botanical extracts; Hp H, *Hypericum perforatum* L. (St. John’s wort, herb); Sg L, *Solidago gigantea* Ait. (giant goldenrod, leaf); To F, To L, *Taraxacum officinale* (L.) Weber ex F.H. Wigg (common dandelion, flower, leaf); Tp F, *Trifolium pratense* L. (red clover, flower); Ur L, *Urtica dioica* L. (nettle, leaf); Vo R, *Valeriana officinalis* L. (valerian, root).

**Table 6:** Effect of the foliar application of the botanical extracts on the sterols composition (the amount of a single component calculated as a percentage (%) of the whole GC-MS chromatogram area) and the sugar content in cabbage head after harvest (*n* = 3, mean ± SD).

Group	Sterols		Sugars	
	Campesterol, TMS Derivative	β-Sitosterol, TMS Derivative	Reducing	Total
	%		g·100 g^−1^ FW	
RT, min	26.383	27.733	-	-
RI_exp	2684	2785	-	-
RI_lit	2689	2789	-	-
C	18.7 ± 70.56	81.23 ± 0.56	3.82 ± 0.18	4.62 ± 0.10 b
CF	19.17 ± 0.38	80.83 ± 0.38	3.30 ± 0.26 c	3.53 ± 0.31 a
CB	18.60 ± 0.50	81.40 ± 0.50	4.10 ± 0.16 b	4.25 ± 0.29
Hp H UAE	19.11 ± 0.18	80.89 ± 0.18	5.64 ± 0.13 a,b,c	5.90 ± 0.16 a,b,c
Hp H MH	20.17 ± 0.78	79.83 ± 0.78	4.74 ± 0.21 a,b	5.20 ± 0.12 b,c
Sg L UAE	19.03 ± 0.29	80.97 ± 0.29	4.00 ± 0.12	4.10 ± 0.08
Sg L MH	20.25 ± 0.51	79.75 ± 0.51	4.75 ± 0.07 a,b	4.83 ± 0.09 b
To F UAE	20.63 ± 0.49	79.37 ± 0.49	3.90 ± 0.28	4.00 ± 0.20
To F MH	19.23 ± 0.39	80.77 ± 0.39	5.70 ± 0.24 a,b,c	5.82 ± 0.27 a,b,c
To L UAE	19.62 ± 0.49	80.38 ± 0.49	4.40 ± 0.08 b	4.60 ± 0.11 b
To L MH	19.29 ± 0.39	80.71 ± 0.39	4.48 ± 0.27 b	4.53 ± 0.14 b
Tp F UAE	19.79 ± 0.83	80.21 ± 0.83	3.42 ± 0.14	3.90 ± 0.24
Tp F MH	19.00 ± 0.55	81.00 ± 0.55	4.20 ± 0.29 b	4.55 ± 0.23 b
Ur L UAE	19.70 ± 0.66	80.30 ± 0.66	4.00 ± 0.26	4.60 ± 0.29 b
Ur L MH	19.36 ± 0.36	80.64 ± 0.36	3.60 ± 0.18	3.91 ± 0.17
Vo R UAE	19.19 ± 0.76	80.81 ± 0.76	2.62 ± 0.27 a,c	4.20 ± 0.22
Vo R MH	20.66 ± 0.74 c	79.34 ± 0.74 c	4.42 ± 0.22 b	4.55 ± 0.16 b

Abbreviations: RT, retention time; RI, retention indices; RI_lit, retention indices according to NIST [29]; RI_exp, retention indices based on experiments. Statistically significant differences (*p* < 0.05) (a) between the control group (C) and the botanical extracts; (b) between the formulation (CF) and the botanical extracts; (c) between commercial biostimulant (CB) and the botanical extracts; Hp H, *Hypericum perforatum* L. (St. John’s wort, herb); Sg L, *Solidago gigantea* Ait. (giant goldenrod, leaf); To F, To L, *Taraxacum officinale* (L.) Weber ex F.H. Wigg (common dandelion, flower, leaf); Tp F, *Trifolium pratense* L. (red clover, flower); Ur L, *Urtica dioica* L. (nettle, leaf); Vo R, *Valeriana officinalis* L. (valerian, root).

**Table 7:** Effect of the foliar application of the botanical extracts on the glucosinolates composition (mg·100 g^−1^ and/or the amount of a single component calculated as a percentage (%) of the whole LC-MS chromatogram area) of cabbage head after harvest (*n* = 3, mean ± SD).

Group	Gluconapin	Glucoiberin	Singrin		Glucoraphanin	Glucobrassicanapin	Gluconasturtin	Glucobrassicin	Neoglucobrassicin	
Retention time, min	2.63	3.39	4.33		4.66	12.23	13.05	21.32	23.09	
Parent ion	372	422	358		436	386	422	447	477	
Fragment ion	97	97	75		97	97	97	97	97	
Collision energy, V	26	26	31		25	25	27	26	24	
Unit	%	%	%	mg·100 g^−1^	%	%	%	%	%	mg·100 g^−1^
C	7.23 ± 0.20	8.05 ± 0.63	16.51 ± 0.38	27.68 ± 0.64	13.75 ± 0.18	6.60 ± 0.22	6.50 ± 0.22	14.01 ± 0.09	27.34 ± 0.25	35.56 ± 0.32
CF	7.47 ± 0.13	8.60 ± 0.26	15.81 ± 0.28	26.50 ± 0.47	13.45 ± 0.12	6.28 ± 0.13	6.46 ± 0.07	14.70 ± 0.29	27.24 ± 0.11	35.42 ± 0.14
CB	7.35 ± 0.24	9.05 ± 0.14	15.79 ± 0.33	26.47 ± 0.55	13.38 ± 0.08	6.47 ± 0.11	6.47 ± 0.20	14.33 ± 0.44	27.17 ± 0.07	35.34 ± 0.09
Hp H UAE	7.35 ± 0.25	8.08 ± 0.75	16.02 ± 0.22	26.86 ± 0.38	13.74 ± 0.15	6.77 ± 0.15	6.47 ± 0.27	14.33 ± 0.27	27.24 ± 0.15	35.42 ± 0.20
Hp H MH	7.40 ± 0.19	8.95 ± 0.09	15.81 ± 0.42	26.50 ± 0.71	13.43 ± 0.10	6.52 ± 0.08	6.37 ± 0.14	14.26 ± 0.55	27.27 ± 0.08	35.46 ± 0.10
Sg L UAE	7.38 ± 0.19	8.18 ± 0.57	16.11 ± 0.22	27.01 ± 0.38	13.62 ± 0.25	6.65 ± 0.17	6.55 ± 0.34	14.13 ± 0.15	27.38 ± 0.26	35.61 ± 0.33
Sg L MH	7.46 ± 0.10	8.57 ± 0.13	15.93 ± 0.25	26.71 ± 0.42	13.56 ± 0.16	6.45 ± 0.07	6.47 ± 0.26	14.35 ± 0.43	27.20 ± 0.15	35.38 ± 0.19
To F UAE	7.38 ± 0.26	8.41 ± 0.36	15.82 ± 0.36	26.52 ± 0.60	13.53 ± 0.16	6.64 ± 0.08	6.60 ± 0.23	14.32 ± 0.38	27.30 ± 0.24	35.51 ± 0.31
To F MH	7.48 ± 0.22	8.93 ± 0.11	15.81 ± 0.40	26.50 ± 0.68	13.43 ± 0.11	6.56 ± 0.08	6.34 ± 0.17	14.17 ± 0.68	27.27 ± 0.09	35.46 ± 0.11
To L UAE	7.36 ± 0.22	8.10 ± 0.66	16.02 ± 0.24	26.86 ± 0.41	13.80 ± 0.13	6.78 ± 0.20	6.62 ± 0.29	14.15 ± 0.08	27.17 ± 0.13	35.34 ± 0.18
To L MH	7.36 ± 0.13	8.80 ± 0.12	15.90 ± 0.29	26.65 ± 0.49	13.43 ± 0.09	6.47 ± 0.08	6.46 ± 0.14	14.37 ± 0.53	27.22 ± 0.07	35.40 ± 0.10
Tp F UAE	7.39 ± 0.17	8.12 ± 0.30	15.76 ± 0.39	26.43 ± 0.66	13.56 ± 0.26	6.66 ± 0.24	6.81 ± 0.26	14.40 ± 0.13	27.30 ± 0.20	35.51 ± 0.27
Tp F MH	7.46 ± 0.19	9.03 ± 0.15	15.79 ± 0.43	26.48 ± 0.73	13.40 ± 0.07	6.54 ± 0.17	6.24 ± 0.15	14.31 ± 0.63	27.23 ± 0.11	35.41 ± 0.14
Ur L UAE	7.36 ± 0.18	8.17 ± 0.42	15.81 ± 0.41	26.51 ± 0.69	13.69 ± 0.19	6.78 ± 0.07	6.40 ± 0.19	14.45 ± 0.42	27.33 ± 0.17	35.54 ± 0.23
Ur L MH	7.39 ± 0.17	8.27 ± 0.39	16.04 ± 0.18	26.89 ± 0.30	13.58 ± 0.22	6.45 ± 0.13	6.68 ± 0.23	14.18 ± 0.14	27.40 ± 0.13	35.63 ± 0.18
Vo R UAE	7.44 ± 0.22	8.14 ± 0.59	16.25 ± 0.21	27.24 ± 0.36	13.69 ± 0.16	6.26 ± 0.14	6.72 ± 0.22	14.07 ± 0.12	27.43 ± 0.35	35.67 ± 0.45
Vo R MH	7.39 ± 0.11	8.63 ± 0.25	15.84 ± 0.29	26.56 ± 0.48	13.39 ± 0.15	6.50 ± 0.06	6.54 ± 0.30	14.34 ± 0.28	27.36 ± 0.17	35.58 ± 0.22

Abbreviations: Hp H, *Hypericum perforatum* L. (St. John’s wort, herb); Sg L, *Solidago gigantea* Ait. (giant goldenrod, leaf); To F, To L, *Taraxacum officinale* (L.) Weber ex F.H. Wigg (common dandelion, flower, leaf); Tp F, *Trifolium pratense* L. (red clover, flower); Ur L, *Urtica dioica* L. (nettle, leaf); Vo R, *Valeriana officinalis* L. (valerian, root).

## Data Availability

Not applicable.

## Supplementary Materials

The following are available online, Figure S1. The weather conditions during the field experiments. Table S1. Effect of the foliar application of the botanical extracts on the L, a, b values of white head cabbage leaves collected seven days after the second foliar application (*n* = 16, mean ± SD). Figure S2. Effect of the foliar application of the botanical extracts on the SPAD values of white head cabbage leaves collected seven days after the second foliar application (*n* = 16). Figure S3a. Effect of the foliar application of the botanical extracts on the total phenolic compounds of outer leaves after the second spraying (*n* = 4). Figure S3b. Effect of the foliar application of the botanical extracts on the total phenolic compounds of white head cabbage (*n* = 4). Table S2a. Effect of the foliar application of the botanical extracts on the macroelements content of white head cabbage (*n* = 3, mean ± SD). Table S2b. Effect of the foliar application of the botanical extracts on the microelements and toxic elements content of white head cabbage (*n* = 3, mean ± SD). Table S3. Effect of the foliar application of the botanical extracts on the volatile compounds profile (the amount of a single component calculated as a percentage (%) of the whole GC-MS chromatogram area) of white head cabbage (*n* = 3, mean ± SD). Table S4. Effect of the foliar application of the botanical extracts on the fatty acids composition (the amount of a single component calculated as a percentage (%) of the whole GC-MS chromatogram area) of white head cabbage (*n* = 3, mean ± SD). Table S5. Effect of the foliar application of the botanical extracts on the sterols composition (the amount of a single component calculated as a percentage (%) of the whole GC-MS chromatogram area) of radish roots (*n* = 3, mean ± SD).

## Sample Availability

Samples of the compounds are not available from the authors.

## References

[1] Milner J.A. (2000). Functional foods: The US perspective. Am. J. Clin. Nutr..

[2] Gul K., Singh A.K., Jabeen R. (2016). Nutraceuticals and functional foods: The foods for the future forld. Crit. Rev. Food Sci. Nutr..

[3] Banerjee E.R. (2017). Nutraceuticals—Prophylactic and therapeutic role of functional food in health. Perspectives in Translational Research in Life Sciences and Biomedicine: Translational Outcomes Research in Life Sciences and Translational Medicine.

[4] Del Castillo M.D., Iriondo-DeHond A., Martirosyan D.M. (2018). Annals of nutrition & food science are functional foods essential for sustainable health?. Ann. Nutr. Food Sci..

[5] Banerjee P., Ray D.P. (2019). Functional food: A brief overview. Int. J. Bioresour. Sci..

[6] Domínguez Díaz L., Fernández-Ruiz V., Cámara M. (2020). The frontier between nutrition and pharma: The international regulatory framework of functional foods, food supplements and nutraceuticals. Crit. Rev. Food Sci. Nutr..

[7] Kumar K., Yadav A.N., Kumar V., Vyas P., Dhaliwal H.S. (2017). Food waste: A potential bioresource for extraction of nutraceuticals and bioactive compounds. Bioresour. Bioprocess..

[8] Arora S., Ranvir S. (2019). Phytochemicals: Benefits, concerns and challenges. Book on Advancement in Functional Food Ingredients.

[9] Nijhawan P., Behl T. (2020). Nutraceuticals in the management of obesity. Obes. Med..

[10] Cencic A., Chingwaru W. (2010). The role of functional foods, nutraceuticals, and food supplements in intestinal health. Nutrients.

[11] Télessy I.G. (2019). Nutraceuticals. The Role of Functional Food Security in Global Health.

[12] Barberis S.E., Origone A.L., Adaro M.O., Bersi G. (2018). Bioactive peptides as functional food ingredients. Role of Materials Science in Food Bioengineering, Handbook of Food Bioengineering.

[13] Varzakas T., Zakynthinos G., Verpoort F. (2016). Plant food residues as a source of nutraceuticals and functional foods. Foods.

[14] Rao K.S. (2018). Safety assessment of nutraceuticals. Biol. Eng. Med. Sci. Rep..

[15] Gulati O.P., Ottaway P.B., Jennings S., Coppens P., Gulati N. (2019). Botanical nutraceuticals (food supplements and fortified and functional foods) and novel foods in the EU, with a main focus on legislative controls on safety aspects. Nutraceutical and Functional Food Regulations in the United States and around the World.

[16] Jaddu S., Katam S. (2018). Hub of health: Nutraceuticals and functional foods. J. Pharmacogn. Phytochem..

[17] Kaur S., Kumar M., Pandit K., Kumar A., Kaur S. (2019). Potential health benefits of nutraceuticals for human health. Environmental Contaminants and Natural Products.

[18] Durazzo A., Lucarini M., Santini A. (2020). Nutraceuticals in human health. Foods.

[19] Singh S., Razak M.A., Sangam S.R., Viswanath B., Begum P.S., Rajagopal S. (2018). The impact of functional food and nutraceuticals in health. Therapeutic Foods.

[20] Isidoro C. (2020). Nutraceuticals and diet in human health and disease. The special issue at a glance. J. Tradit. Complement. Med..

[21] Povero G., Mejia J.F., Di Tommaso D., Piaggesi A., Warrior P. (2016). A systematic approach to discover and characterize natural plant biostimulants. Front. Plant Sci..

[22] Rouphael Y., Colla G. (2020). Toward a sustainable agriculture through plant biostimulants: From experimental data to practical applications. Agronomy.

[23] Kocira A., Kocira S., Świeca M., Złotek U., Jakubczyk A., Kapela K. (2017). Effect of foliar application of a nitrophenolate–based biostimulant on the yield and quality of two bean cultivars. Sci. Hortic..

[24] Kocira S., Szparaga A., Findura P., Treder K. (2020). Modification of yield and fiber fractions biosynthesis in *Phaseolus*
*vulgaris* by treatment with biostimulants containing amino acids and seaweed extract. Agronomy.

[25] Du Jardin P. (2012). The Science of Plant Biostimulants—A bibliographic analysis. Contract 30-CEO455515/00-96.

[26] Ricci M., Tilbury L., Daridon B., Sukalac K. (2019). General principles to justify plant biostimulant claims. Front. Plant Sci..

[27] Kocira S., Szparaga A., Hara P., Treder K., Findura P., Bartoš P., Filip M. (2020). Biochemical and economical effect of application biostimulants containing seaweed extracts and amino acids as an element of agroecological management of bean cultivation. Sci. Rep..

[28] Godlewska K., Pacyga P., Michalak I., Biesiada A., Szumny A., Pachura N., Piszcz U. (2020). Field-scale evaluation of botanical extracts effect on the yield, chemical composition and antioxidant activity of celeriac (*Apium graveolens* L. var. *rapaceum*). Molecules.

[29] Godlewska K., Biesiada A., Michalak I., Pacyga P. (2019). The effect of plant-derived biostimulants on white head cabbage seedlings grown under controlled conditions. Sustainability.

[30] Godlewska K., Biesiada A., Michalak I., Pacyga P. (2020). The effect of botanical extracts obtained through ultrasound-assisted extraction on white head cabbage (*Brassica oleracea* L. var. *capitata* L.) seedlings grown under controlled conditions. Sustainability.

[31] Arnon D.I. (1949). Copper enzymes in isolated chloroplasts. Polyphenoloxidase in *Beta vulgaris*. Plant Physiol..

[32] Bieniek A. (2012). Yield, morphology and biological value of fruits of *Actinidia arguta* and *Actinidia purpurea* and some of their hybrid cultivars grown in north-eastern Poland. Acta Sci. Pol. Hortorum Cultus.

[33] Krężel J., Kołota E. (2014). Source of nitrogen affects the yield and quality of spinach cultivars grown for autumn harvest. Acta Agric. Scand. Sect. B Soil Plant Sci..

[34] PN-90/A-75101/11 (1990). Fruit and Vegetable Products—Preparation of Samples and Testing Methods—Determination of Ascorbic Acid Content (In Polish—Przetwory Owocowe i Warzywne. Przygotowanie Próbek i Metody Badań Fizyko-Chemicznych. Oznaczenie Zawartości Witaminy C).

[35] Jałoszyński K., Figiel A., Wojdyło A. (2008). Drying kinetics and antioxidant activity of oregano. Acta Agrophys..

[36] Yen G.C., Chen H.Y. (1995). Antioxidant activity of various tea extracts in relation to their antimutagenicity. J. Agric. Food Chem..

[37] Re R., Pellegrini N., Proteggente A., Pannala A., Yang M., Rice-Evans C. (1999). Antioxidant activity applying and improved ABTS radical cation decolorization assay. Free Radic. Biol. Med..

[38] Almeida M.M.B., de Sousa P.H.M., Arriaga Â.M.C., do Prado G.M., de Carvalho Magalhães C.E., Maia G.A., de Lemos T.L.G. (2011). Bioactive compounds and antioxidant activity of fresh exotic fruits from northeastern Brazil. Food Res. Int..

[39] Benzie I.F.F., Strain J.J. (1996). The ferric reducing ability of plasma (FRAP) as a measure of “Antioxidant Power”: The FRAP assay. Anal. Biochem..

[40] Nowosielski O. (1988). Principles of Preparation of Fertilizing Recommendations in Horticulture (In Polish; Zasady Opracowywania Zaleceń Nawozowych w Ogrodnictwie).

[41] Butters B., Chenery E.M. (1959). A rapid method for the determination of total sulphur in soils and plants. Analyst.

[42] Bielecki K., Kulczycki G. (2012). Modification of Butters-Chenery method for determination of total sulfur in plants and soil. Przem. Chem..

[43] Jones J.B. (2001). Laboratory Guide for Conducting Soil Tests and Plant Analysis.

[44] Calín-Sánchez Á., Figiel A., Lech K., Szumny A., Martínez-Tomé J., Carbonell-Barrachina Á.A. (2015). Dying methods affect the aroma of *Origanum majorana* L. analyzed by GC-MS and descriptive sensory analysis. Ind. Crops Prod..

[45] Folch J., Lees M., Sloane Stanley G.H. (1957). A simple method for the isolation and purification of total lipides from animal tissues. J. Biol. Chem..

[46] Maslak E., Buczek E., Szumny A., Szczepnski W., Franczyk-Zarow M., Kopec A., Chlopicki S., Leszczynska T., Kostogrys R.B. (2015). Individual CLA isomers, c9t11 and t10c12, prevent excess liver glycogen storage and inhibit lipogenic genes expression induced by high-fructose diet in rats. BioMed Res. Int..

[47] Gholami Zali A., Ehsanzadeh P., Szumny A., Matkowski A. (2018). Genotype-specific response of Foeniculum vulgare grain yield and essential oil composition to proline treatment under different irrigation conditions. Ind. Crops Prod..

[48] Abidi S.L. (2001). Chromatographic analysis of plant sterols in foods and vegetable oils. J. Chromatogr. A.

[49] Baenas N., Marhuenda J., García-Viguera C., Zafrilla P., Moreno D.A. (2019). Influence of cooking methods on glucosinolates and isothiocyanates content in novel cruciferous foods. Foods.

[50] PN-90/A-75101/07 (1990). Fruit and Vegetable Products—Preparation of Samples and Testing Methods. Determination of Sugars and Sugar-Free Extract (In Polish—Przetwory Owocowe i Warzywne. Przygotowanie Próbek i Metody Badań. Oznaczanie Zawartości Cukrów i Ekstraktu Bezcukrowego.

[51] Chin H.-W., Lindsay R.C. (1993). Volatile sulfur compounds formed in disrupted tissues of different cabbage cultivars. J. Food Sci..

[52] Hallmann E., Kazimierczak R., Marszałek K., Drela N., Kiernozek E., Toomik P., Matt D., Luik A., Rembiałkowska E. (2017). The nutritive value of organic and conventional white cabbage (*Brassica oleracea* L. var. *capitata*) and anti-apoptotic activity in gastric adenocarcinoma cells of sauerkraut juice produced therof. J. Agric. Food Chem..

[53] www.globaltrademag.com.

[54] Kosson R., Felczyński K., Szwejda-Grzybowska J., Grzegorzewska M., Tuccio L., Agati G., Kaniszewski S. (2017). Nutritive value of marketable heads and outer leaves of white head cabbage cultivated at different nitrogen rates. Acta Agric. Scand. Sect. B Soil Plant Sci..

[55] Ali M., Cheng Z.H., Hayat S., Ahmad H., Ghani M.I., LIU T. (2019). Foliar spraying of aqueous garlic bulb extract stimulates growth and antioxidant enzyme activity in eggplant (*Solanum melongena* L.). J. Integr. Agric..

[56] Elzaawely A.A., Ahmed M.E., Maswada H.F., Al-Araby A.A., Xuan T.D. (2018). Growth traits, physiological parameters and hormonal status of snap bean (*Phaseolus vulgaris* L.) sprayed with garlic cloves extract. Arch. Agron. Soil Sci..

[57] Basra S.M.A., Lovatt C.J. (2016). Exogenous applications of moringa leaf extract and cytokinins improve plant growth, yield, and fruit quality of cherry tomato. Horttechnology.

[58] Mazrou R.M. (2019). Moringa leaf extract application as a natural biostimulant improves the volatile oil content, radical scavenging activity and total phenolics of coriander. J. Med. Plants Stud..

[59] Khan S., Basra S.M.A., Afzal I., Wahid A. (2017). Screening of moringa landraces for leaf extract as biostimulant in wheat. Int. J. Agric. Biol..

[60] Merwad A.R.M.A. (2018). Using *Moringa oleifera* extract as biostimulant enhancing the growth, yield and nutrients accumulation of pea plants. J. Plant Nutr..

[61] Mona M.A. (2013). The potential of *Moringa oleifera* extract as a biostimulant in enhancing the growth, biochemical and hormonal contents in rocket (*Eruca vesicaria* subsp. *sativa*) plants. Int. J. Plant Physiol. Biochem..

[62] Rady M.M., Desoky E.S.M., Elrys A.S., Boghdady M.S. (2019). Can licorice root extract be used as an effective natural biostimulant for salt-stressed common bean plants?. S. Afr. J. Bot..

[63] Babilie R., Jbour M., Trabi B.A. (2015). Effect of foliar spraying with licorice root and seaweed extractson growth and seed production of onion (*Allium cepa* L.). Int. J. Chem. Tech. Res..

[64] Thanaa S.M., Nabila E.K., Abou Rayya M.S., Eisa R.A. (2016). Response of Nonpareil seedlings almond to foliar application of liquorice root extract and bread yeast suspend under South Sinai conditions. J. Innov. Pharm. Biol. Sci..

[65] El-Azim A., Khater W.M., And Badawy R.M.R. (2017). Effect of bio-fertilization and different licorice extracts on growth and productivity of *Foeniculum vulgare*, Mill. plant. Middle East J. Agric. Res..

[66] Ertani A., Pizzeghello D., Francioso O., Tinti A., Nardi S. (2016). Biological activity of vegetal extracts containing phenols on plant metabolism. Molecules.

[67] Ganagi T.I., Jagadeesh K.S. (2018). Effect of spraying Lantana fermented extract on growth and yield of green gram (*Vigna radiata* L.) in pots. Int. J. Curr. Microbiol. Appl. Sci..

[68] Calvo P., Nelson L., Kloepper J.W. (2014). Agricultural uses of plant biostimulants. Plant Soil.

[69] Yakhin O.I., Lubyanov A.A., Yakhin I.A., Brown P.H. (2017). Biostimulants in plant science: A global perspective. Front. Plant Sci..

[70] Di Mola I., Ottaiano L., Cozzolino E., Senatore M., Giordano M., El-nakhel C., Sacco A., Rouphael Y., Colla G. (2019). Plant-Based biostimulants influence the agronomical, physiological, and qualitative responses of baby rocket leaves under diverse nitrogen conditions. Plants.

[71] Dipak Kumar H., Aloke P. (2020). Role of biostimulant formulations in crop production: An overview. Int. J. Agric. Sci. Vet. Med..

[72] Lee S.K., Kader A.A. (2000). Preharvest and postharvest factors influencing vitamin C content of horticultural crops. Postharvest Biol. Technol..

[73] Ajibola V.O., Babatunde O.A., Suleiman S. (2009). The effect of storage method on the vitamin C content in some tropical fruit juices. Trends Appl. Sci. Res..

[74] Abbasi A.M., Shah M.H., Khan M.A. (2015). Phytochemicals and nutraceuticals. Wild Edible Vegetables of Lesser Himalayas: Ethnobotanical and Nutraceutical Aspects.

[75] Gallie D.R. (2013). L-Ascorbic acid: A multifunctional molecule supporting plant growth and development. Scientifica.

[76] Paciolla C., Fortunato S., Dipierro N., Paradiso A., De Leonardis S., Mastropasqua L., de Pinto M.C. (2019). Vitamin C in plants: From functions to biofortification. Antioxidants.

[77] Frei B., Birlouez-Aragon I., Lykkesfeldt J. (2012). Authors’ perspective: What is the optimum intake of vitamin C in humans?. Crit. Rev. Food Sci. Nutr..

[78] Carr A.C., Lykkesfeldt J. (2021). Discrepancies in global vitamin C recommendations: A review of RDA criteria and underlying health perspectives. Crit. Rev. Food Sci. Nutr..

[79] Miceli A., Miceli C. (2014). Effect of nitrogen fertilization on the quality of swiss chard at harvest and during storage as minimally processed produce. J. Food Qual..

[80] Thanaa S., Kassim N., AbouRayya M., Abdalla A. (2017). Influence of foliar application with moringa (*Moringa oleifera* L.) leaf extract on yield and fruit quality of Hollywood plum cultivar. J. Hortic..

[81] Donno D., Beccaro G.L., Mellano M.G., Canterino S., Cerutti A.K., Bounous G. (2013). Improving the nutritional value of kiwifruit with the application of agroindustry waste extracts. J. Appl. Bot. Food Qual..

[82] Giordano M., El-Nakhel C., Caruso G., Cozzolino E., De Pascale S., Kyriacou M.C., Colla G., Rouphael Y. (2020). Stand-alone and combinatorial effects of plant-based biostimulants on the production and leaf quality of perennial wall rocket. Plants.

[83] Koch W. (2019). Dietary polyphenols—Important non-nutrients in the prevention of chronic noncommunicable. Nutrients.

[84] Del Bo C., Bernardi S., Marino M., Porrini M., Tucci M., Guglielmetti S., Cherubini A., Carrieri B., Kirkup B., Kroon P. (2019). Systematic review on polyphenol intake and health outcomes: Is there sufficient evidence to define a health-promoting polyphenol-rich dietary pattern?. Nutrients.

[85] Scalbert A., Williamson G. (2000). Dietary intake and bioavailability of polyphenols. J. Nutr..

[86] Ovaskainen M.-L., Törrönen R., Koponen J.M., Sinkko H., Hellström J., Reinivuo H., Mattila P. (2008). Dietary intake and major food sources of polyphenols in finnish adults. J. Nutr..

[87] Wisnuwardani R.W., De Henauw S., Androutsos O., Forsner M., Gottrand F., Huybrechts I., Knaze V., Kersting M., Le Donne C., Marcos A. (2019). Estimated dietary intake of polyphenols in European adolescents: The HELENA study. Eur. J. Nutr..

[88] Fraga C.G., Croft K.D., Kennedy D.O., Tomás-Barberán F.A. (2019). The effects of polyphenols and other bioactives on human health. Food Funct..

[89] Witkowska A.M., Zujko M.E., Waśkiewicz A., Terlikowska K.M., Piotrowski W. (2015). Comparison of various databases for estimation of dietary polyphenol intake in the population of polish adults. Nutrients.

[90] Šamec D., Pavlović I., Salopek-Sondi B. (2017). White cabbage (*Brassica oleracea* var. *capitata* f. *alba*): Botanical, phytochemical and pharmacological overview. Phytochem. Rev..

[91] Abbas M., Saeed F., Anjum F.M., Afzaal M., Tufail T., Bashir M.S., Ishtiaq A., Hussain S., Suleria H.A.R. (2017). Natural polyphenols: An overview. Int. J. Food Prop..

[92] Mohamed M.H., Badr E.A., Sadak M.S., Khedr H.H. (2020). Effect of garlic extract, ascorbic acid and nicotinamide on growth, some biochemical aspects, yield and its components of three faba bean (*Vicia faba* L.) cultivars under sandy soil conditions. Bull. Natl. Res. Cent..

[93] Findura P., Kocira S., Hara P., Pawłowska A., Szparaga A., Kangalov P. (2020). Extracts from *Artemisia vulgaris* L. In potato cultivation—preliminary research on biostimulating effect. Agriculture.

[94] Bulgari R., Morgutti S., Cocetta G., Negrini N., Farris S., Calcante A., Spinardi A., Ferrari E., Mignani I., Oberti R. (2017). Evaluation of borage extracts as potential biostimulant using a phenomic, agronomic, physiological, and biochemical approach. Front. Plant Sci..

[95] Pardo-García A.I., Martínez-Gil A.M., Cadahía E., Pardo F., Alonso G.L., Salinas M.R. (2014). Oak extract application to grapevines as a plant biostimulant to increase wine polyphenols. Food Res. Int..

[96] Teklić T., Parađiković N., Špoljarević M., Zeljković S., Lončarić Z., Lisjak M. (2020). Linking abiotic stress, plant metabolites, biostimulants and functional food. Ann. Appl. Biol..

[97] Graziani G., Ritieni A., Cirillo A., Cice D., Di Vaio C. (2020). Effects of biostimulants on annurca fruit quality and potential nutraceutical compounds at harvest and during storage. Plants.

[98] Heimler D., Romani A., Ieri F. (2017). Plant polyphenol content, soil fertilization and agricultural management: A review. Eur. Food Res. Technol..

[99] Ismail A., Marjan Z.M., Foong C.W. (2004). Total antioxidant activity and phenolic content in selected vegetables. Food Chem..

[100] Rashid N., Wahid A., Basra S.M.A., Arfan M. (2020). Foliar spray of moringa leaf extract, sorgaab, hydrogen peroxide and ascorbic acid improve leaf physiological and seed quality traits of quinoa (*Chenopodium quinoa*) under terminal heat stress. Int. J. Agric. Biol..

[101] Ertani A., Schiavon M., Muscolo A., Nardi S. (2012). Alfalfa plant-derived biostimulant stimulate short-term growth of salt stressed Zea mays L. plants. Plant Soil.

[102] Rahman M., Mukta J.A., Sabir A.A., Gupta D.R., Mohi-Ud-Din M., Hasanuzzaman M., Miah M.G., Rahman M., Islam M.T. (2018). Chitosan biopolymer promotes yield and stimulates accumulation of antioxidants in strawberry fruit. PLoS ONE.

[103] Drobek M., Frąc M., Cybulska J. (2019). Plant biostimulants: Importance of the quality and yield of horticultural crops and the improvement of plant tolerance to abiotic stress—A review. Agronomy.

[104] Szczepanek M., Pobereżny J., Wszelaczyńska E., Gościnna K. (2020). Effect of biostimulants and storage on discoloration potential of carrot. Agronomy.

[105] Mirecki N., Hić Z.S., Šunić L., Rukie A. (2015). Nitrate content in carrot, celeriac and parsnip at harvest time and during prolonged cold storage. Fresenius Environ. Bull..

[106] Hmelak Gorenjak A., Cencič A. (2013). Nitrate in vegetables and their impact on human health. A review. Acta Aliment..

[107] Bahadoran Z., Mirmiran P., Jeddi S., Azizi F., Ghasemi A., Hadaegh F. (2016). Nitrate and nitrite content of vegetables, fruits, grains, legumes, dairy products, meats and processed meats. J. Food Compos. Anal..

[108] Colla G., Kim H.J., Kyriacou M.C., Rouphael Y. (2018). Nitrate in fruits and vegetables. Sci. Hortic..

[109] Mozafar A. (1996). Decreasing the NO_3_ and increasing the vitamin C contents in spinach by a nitrogen deprivation method. Plant Foods Hum. Nutr..

[110] Hanif R., Iqbal Z., Iqbal M., Hanif S., Rasheed M. (2006). Use of vegetables as nutritional food: Role in human health. J. Agric. Biol. Sci..

[111] Soetan K.O., Olaiya C.O., Oyewole O.E. (2010). The importance of mineral elements for humans, domestic animals and plants: A review. Afr. J. Food Sci..

[112] Rehman H.U., Alharby H.F., Alzahrani Y., Rady M.M. (2018). Magnesium and organic biostimulant integrative application induces physiological and biochemical changes in sunflower plants and its harvested progeny on sandy soil. Plant Physiol. Biochem..

[113] Halpern M., Bar-Tal A., Ofek M., Minz D., Muller T., Yermiyahu U. (2015). The Use of Biostimulants for Enhancing Nutrient Uptake. Advances in Agronomy.

[114] De Pascale S., Rouphael Y., Colla G. (2017). Plant biostimulants: Innovative tool for enhancing plant nutrition in organic farming. Eur. J. Hortic. Sci..

[115] Szczepanek M., Wilczewski E., Pobereżny J., Wszelaczyńska E., Keutgen A., Ochmian I. (2015). Effect of biostimulants and storage on the content of macroelements in storage roots of carrot. J. Elementol..

[116] Dziugieł T., Wadas W. (2020). Effect of plant biostimulants on macronutrient content in early crop potato tubers. Agronomy.

[117] Figueiredo A.C., Barroso J.G., Pedro L.G., Scheffer J.J.C. (2008). Factors affecting secondary metabolite production in plants: Volatile components and essential oils. Flavour Fragr. J..

[118] Djilani A., Dicko A. (2012). The therapeutic benefits of essential oils. Nutr. Well-Being Health.

[119] Tongnuanchan P., Benjakul S. (2014). Essential oils: Extraction, bioactivities, and their uses for food preservation. J. Food Sci..

[120] Rehman R., Hanif M.A., Mushtaq Z., Al-Sadi A.M. (2016). Biosynthesis of essential oils in aromatic plants: A review. Food Rev. Int..

[121] Stevović S., Ćalić-Dragosavac D., Mikovilović V.S., Zdravković-Korać S., Milojević J., Cingel A. (2011). Correlation between environment and essential oil production in medical plants. Adv. Environ. Biol..

[122] Abbas F., Ke Y., Yu R., Yue Y., Amanullah S., Jahangir M.M., Fan Y. (2017). Volatile terpenoids: Multiple functions, biosynthesis, modulation and manipulation by genetic engineering. Planta.

[123] Akbar S. (2020). Handbook of 200 Medicinal Plants.

[124] He M., Qin C.X., Wang X., Ding N.Z. (2020). Plant unsaturated fatty acids: Biosynthesis and regulation. Front. Plant Sci..

[125] Li M.Y., Feng K., Hou X.L., Jiang Q., Xu Z.S., Wang G.L., Liu J.X., Wang F., Xiong A.S. (2020). The genome sequence of celery (*Apium graveolens* L.), an important leaf vegetable crop rich in apigenin in the Apiaceae family. Hortic. Res..

[126] Yang R., Xue L., Zhang L., Wang X., Qi X., Jiang J., Yu L., Wang X., Zhang W., Zhang Q. (2019). Phytosterol contents of edible oils and their contributions to estimated phytosterol intake in the Chinese diet. Foods.

[127] Tolonen M., Taipale M., Viander B., Pihlava J.M., Korhonen H., Ryhänen E.L. (2002). Plant-derived biomolecules in fermented cabbage. J. Agric. Food Chem..

[128] Cartea M.E., Velasco P. (2008). Glucosinolates in *Brassica* foods: Bioavailability in food and significance for human health. Phytochem. Rev..

[129] Sarıkamış G., Yıldırım A., Alkan D. (2015). Glucosinolates in seeds, sprouts and seedlings of cabbage and black radish as sources of bioactive compounds. Can. J. Plant Sci..

[130] Singh S., Bilashini Devi M. (2015). Vegetables as a potential source of nutraceuticals and phytochemicals: A review. Int. J. Med. Pharm. Sci..

[131] Favela-González K.M., Hernández-Almanza A.Y., De la Fuente-Salcido N.M. (2020). The value of bioactive compounds of cruciferous vegetables (*Brassica*) as antimicrobials and antioxidants: A review. J. Food Biochem..

[132] Ciska E., Kozłowska H. (2001). The effect of cooking on the glucosinolates content in white cabbage. Eur. Food Res. Technol..

[133] Prakash D., Gupta C. (2012). Glucosinolates: The phytochemicals of nutraceutical importance. J. Complement. Integr. Med..

[134] D’Antuono L.F., Elementi S., Neri R. (2007). Sensory attributes, health promoting aspects and new uses of edible brassicaceae. Acta Hortic..

[135] Pandey S., Garg F.C. (2013). Preparation of spiced sauerkraut by using lactic acid bacteria and by natural fermentation. Int. J. Sci. Res..

[136] Halász A., Baráth Á., Holzapfel W.H. (1999). The influence of starter culture selection on sauerkraut fermentation. Eur. Food Res. Technol..

[137] Casadesús A., Pérez-Llorca M., Munné-Bosch S., Polo J. (2020). An enzymatically hydrolyzed animal protein-based biostimulant (Pepton) increases salicylic acid and promotes growth of tomato roots under temperature and nutrient stress. Front. Plant Sci..

[138] Petropoulos S.A., Fernandes Â., Plexida S., Chrysargyris A., Tzortzakis N., Barreira J.C.M., Barros L., Ferreira I.C.F.R. (2020). Biostimulants application alleviates water stress effects on yield and chemical composition of greenhouse green bean (*Phaseolus vulgaris* L.). Agronomy.

[139] Xu L., Trinh H.K., Geelen D. (2020). Biostimulant mode of action: Impact of PBs on molecular level. The Chemical Biology of Plant Biostimulants.

[140] Rouphael Y., Colla G. (2018). Synergistic biostimulatory action: Designing the next generation of plant biostimulants for sustainable agriculture. Front. Plant Sci..

