# Supplementary material for: Effect of Botanical Extracts on the Growth and Nutritional Quality of Field-Grown White Head Cabbage (Brassica oleracea var. capitata)
Source: Molecules. 2021 Apr 1;26(7):1992. doi: 10.3390/molecules26071992 (PMC8037025; doi:10.3390/molecules26071992)
Supplement: Supplementary file 1 [file molecules-26-01992-s001.pdf]

molecules

MDPI

Supplementary

Effect of Botanical Extracts on the Growth and Nutritional Quality of Field-Grown White Head Cabbage (*Brassica oleracea* var. *capitata*)

Katarzyna Godlewska [1,*], Paweł Pacyga [2], Izabela Michalak [3], Anita Biesiada [1], Antoni Szumny [4], Natalia Pachura [4] and Urszula Piszcz [5]

1 Department of Horticulture, Faculty of Life Sciences and Technology, Wrocław University of Environmental and Life Sciences, 50-363 Wrocław, Poland; anita.biesiada@upwr.edu.pl
2 Department of Energy Technologies, Turbines, and Modeling of Heat-Flow Processes, Faculty of Mechanical and Power Engineering, Wrocław University of Science and Technology, 50-370 Wrocław, Poland; pawel.pacyga@pwr.edu.pl
3 Department of Advanced Material Technologies, Faculty of Chemistry, Wrocław University of Science and Technology, 50-372 Wrocław, Poland; izabela.michalak@pwr.edu.pl
4 Department of Chemistry, Faculty of Biotechnology and Food Science, Wrocław University of Environmental and Life Sciences, 50-375 Wrocław, Poland; antoni.szumny@upwr.edu.pl (A.S.); natalia.pachura@upwr.edu.pl (N.P.)
5 Department of Plant Nutrition, The Faculty of Life Sciences and Technology, Wrocław University of Environmental and Life Sciences, 50-357 Wrocław, Poland; urszula.piszcz@upwr.edu.pl
* Correspondence: katarzyna.godlewska@upwr.edu.pl

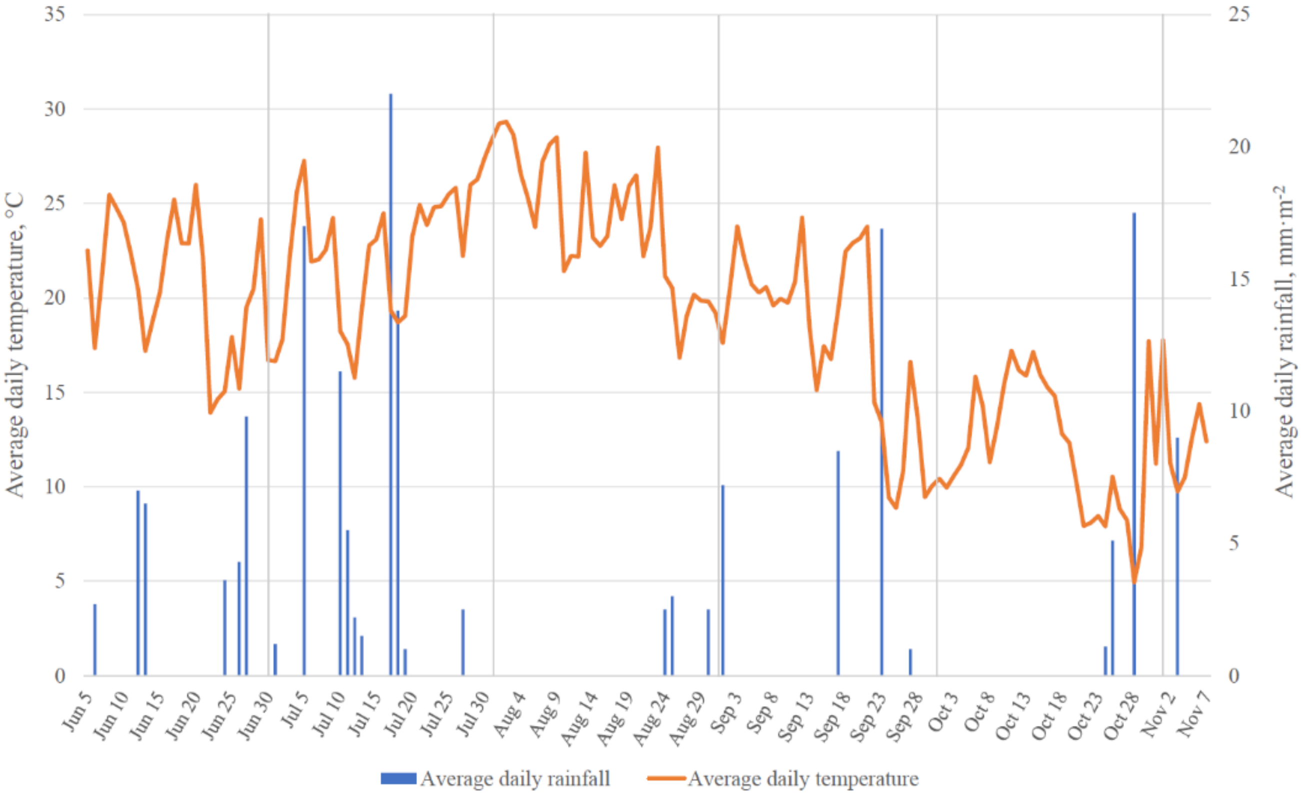

Figure S1. The weather conditions during the field experiments.

Table S1. Effect of the foliar application of the botanical extracts on the volatile compounds profile (the amount of a single component calculated as a percentage (%) of the whole GC-MS chromatogram area) of cabbage head after harvest (N=3, mean ± SD).

Group	Hexanal	Hex-(2*E*)-enal	3-Hexen-1-ol	Isothiocyanate <2-propenyl->	3-Hepten-2-ol	1-Heptanol	Hept-(4*Z*)-enal	2,4-Nonadienol, <(2*E*, 4*E*)>	Methyl allyl di-sulfide	1-Hexanethiol
RT, min	4.55	6.08	6.69	7.17	7.53	7.91	8.06	8.28	8.46	8.53
RI_exp	800	855	864	875	892	898	905	909	914	926
RI_NIST	800	854	856	880	887	895	900	908	920	922
RI_FFNSC	801	850	868	880	887	894	902	906	919	921
RI_Adams	801	855	869	881	888	896	899	909	915	923
C	2.33±0.26 b	0.64±0.11	0.16±0.02	0.91±0.05	0.11±0.02	0.05±0.01 b	0.02±0.01 b	0.03±0.00	0.02±0.01	0.03±0.00
CF	3.65±0.30 a,c	1.26±0.15	0.16±0.02	0.86±0.04	0.14±0.01	0.10±0.01 a	0.07±0.01 a	0.06±0.01 c	0.02±0.01	0.05±0.00
CB	2.75±0.10 b	1.13±0.09	0.16±0.02	0.66±0.07	0.11±0.02	0.09±0.01	0.05±0.00	0.02±0.00 b	0.02±0.01	0.06±0.01
Hp H UAE	2.48±0.12 b	1.26±0.13	0.12±0.00	0.50±0.07 a,b	0.07±0.01	0.07±0.01	0.04±0.00	0.03±0.00	0.01±0.00	0.07±0.01
Hp H MH	2.47±0.24 b	1.01±0.06	0.14±0.03	0.58±0.08 a	0.07±0.01	0.06±0.01	0.08±0.01 a	0.02±0.01 b	0.03±0.00	0.08±0.01
Sg L UAE	2.35±0.06 b	1.01±0.13	0.12±0.03	0.66±0.17	0.10±0.02	0.07±0.01	0.05±0.00	0.05±0.01 c	0.08±0.01 a,b,c	0.09±0.00
Sg L MH	2.66±0.10 b	1.53±0.06 a	0.23±0.02	1.22±0.07 a,b,c	0.12±0.02	0.07±0.01	0.07±0.01 a	0.04±0.01	0.01±0.00	0.03±0.00
To F UAE	3.52±0.25 a,c	1.41±0.10 a	0.18±0.01	0.94±0.09	0.13±0.01	0.09±0.02 a	0.07±0.01 a	0.04±0.01	0.06±0.01 a,b,c	0.09±0.01
To F MH	3.41±0.34 a	0.95±0.06	0.19±0.02	0.66±0.08	0.12±0.03	0.07±0.01	0.10±0.01 a,c	0.04±0.00	0.01±0.00	0.07±0.00
To L UAE	2.64±0.19 b	1.13±0.04	0.12±0.02	0.49±0.04 a,b	0.06±0.00 b	0.07±0.01	0.06±0.00	0.04±0.00	0.05±0.01 a,b,c	0.09±0.04
To L MH	4.25±0.16 a,c	1.21±0.05	0.23±0.00	1.13±0.07 c	0.27±0.05 a,b,c	0.12±0.01 a	0.11±0.01 a,b,c	0.06±0.01 c	0.04±0.01	0.18±0.04 a,b,c
Tp F UAE	3.85±0.06 a,c	1.78±0.55 a,c	0.13±0.02	0.87±0.03	0.05±0.01 b	0.13±0.02 a	0.06±0.01 a	0.03±0.00	0.04±0.01	0.10±0.02 a
Tp F MH	4.20±0.14 a,c	1.35±0.16 a	0.18±0.01	0.74±0.10	0.16±0.02	0.11±0.01 a	0.09±0.00 a,c	0.05±0.01 c	0.01±0.01	0.04±0.00
Ur L UAE	3.09±0.04 a	1.36±0.15 a	0.20±0.04	0.76±0.09	0.11±0.02	0.08±0.01	0.08±0.02 a	0.04±0.01	0.03±0.01	0.10±0.02 a
Ur L MH	3.12±0.11 a	1.40±0.09 a	0.17±0.02	0.86±0.10	0.12±0.01	0.07±0.02	0.06±0.00 a	0.03±0.00	0.01±0.01	0.02±0.00
Vo R UAE	1.42±0.15 a,b,c	0.95±0.10	0.12±0.02	0.52±0.02 a,b	0.07±0.01	0.07±0.02	0.05±0.01	0.03±0.00 b	0.01±0.00	0.02±0.00
Vo R MH	3.34±0.08 a	1.47±0.20 a	0.11±0.02	0.63±0.08	0.13±0.02	0.09±0.00	0.09±0.00 a,c	0.05±0.00 c	0.01±0.00	0.06±0.02

Statistically significant differences ($p < 0.05$) (a) between the control group (C) and the botanical extracts; (b) between the formulation (CF) and the botanical extracts; (c) between commercial biostimulant (CB) and the botanical extracts; Hp H, *Hypericum perforatum* L. (St. John's wort, herb); Sg L, *Solidago gigantea* Ait. (giant goldenrod, leaf); To F, To L, *Taraxacum officinale* (L.) Weber ex F.H. Wigg (common dandelion, flower, leaf); Tp F, *Trifolium pratense* L. (red clover, flower); Ur L, *Urtica dioica* L. (nettle, leaf); Vo R, *Valeriana officinalis* L. (valerian, root).**Table S1 – continuation.** Effect of the foliar application of the botanical extracts on the volatile compounds profile (the amount of a single component calculated as a percentage (%) of the whole GC-MS chromatogram area) of cabbage head after harvest (N=3, mean ± SD).

**Group**	**Pinene <alpha->**	**Butanal, 3-(methylthio)-**	**Hept-(2*E*)-enal**	**Trisulfide, dimethyl-**	**Pinene <beta->**	**Geranyl formate**	**Isothiocyanate <3-bu-tenyl->**	**Octanal**	**Furan, 2-pentyl-**	**Hexenyl acetate <(3Z)->**
RT, min	9.30	10.10	10.42	10.95	11.39	11.67	11.77	11.94	12.29	12.94
RI_exp	932	953	960	970	977	982	983	986	992	1004
RI_NIST	937	953	958	970	979	980	981	989	933	1005
RI_FFNSC	930	950	956	969	978	979	980	983	991	1006
RI_Adams	939	951	954	971	979	983	984	989	994	1005
C	0.01±0.00 b,c	0.07±0.01	0.11±0.01 b	29.24±1.50	0.03±0.00	0.19±0.02	0.10±0.01	0.03±0.01	1.54±0.27 b	0.03±0.00
CF	0.05±0.00 a	0.09±0.00	0.21±0.02 a,c	31.11±0.45	0.08±0.02	0.37±0.03	0.11±0.01	0.05±0.01	2.16±0.12 a	0.07±0.01
CB	0.07±0.01 a	0.04±0.00	0.11±0.01 b	29.38±0.47	0.09±0.01	0.22±0.03	0.14±0.02	0.03±0.01	1.78±0.07	0.09±0.00
Hp H UAE	0.04±0.01 a	0.10±0.01	0.14±0.01	30.70±0.45	0.09±0.02	0.34±0.06	0.10±0.01	0.03±0.01	1.84±0.10	0.07±0.01
Hp H MH	0.05±0.01 a	0.22±0.02 a,b,c	0.13±0.01 b	33.11±0.66 a,c	0.07±0.01	0.32±0.03	0.09±0.02	0.04±0.00	1.55±0.08 b	0.06±0.01
Sg L UAE	0.03±0.00 c	0.29±0.02 a,b,c	0.13±0.02	24.83±1.45 a,b,c	0.05±0.01	0.19±0.04	0.16±0.02	0.04±0.01	1.84±0.24	0.07±0.00
Sg L MH	0.05±0.01 a	0.09±0.01	0.15±0.04	29.29±0.52	0.05±0.01	0.95±0.08 a,b,c	0.26±0.01 a,b,c	0.06±0.01	1.95±0.16	0.06±0.01
To F UAE	0.05±0.01 a	0.24±0.02 a,b,c	0.17±0.04	32.63±0.51 a	0.10±0.03 a	0.44±0.06 a,c	0.19±0.03 a,b	0.06±0.00	1.97±0.06	0.12±0.02 a
To F MH	0.04±0.00 a	0.05±0.00	0.16±0.01	27.79±0.28	0.06±0.01	0.56±0.05 a,c	0.13±0.01	0.05±0.01	1.56±0.06 b	0.07±0.01
To L UAE	0.04±0.01 a,c	0.24±0.06 a,b,c	0.13±0.03 b	25.74±0.51 a,b,c	0.08±0.01	0.20±0.01	0.10±0.02	0.05±0.00	1.80±0.06	0.12±0.02 a
To L MH	0.04±0.01 a	0.16±0.02 a,c	0.23±0.01 a,c	31.53±0.27	0.07±0.01	0.61±0.07 a,b,c	0.16±0.03	0.07±0.01 a,c	2.47±0.16 a,c	0.13±0.03 a
Tp F UAE	0.05±0.01 a	0.15±0.02 c	0.25±0.03 a,c	32.01±0.22	0.08±0.00	0.64±0.08 a,b,c	0.16±0.01	0.08±0.02 a,c	2.14±0.05 a	0.16±0.05 a,b,c
Tp F MH	0.08±0.02 a	0.08±0.02	0.20±0.02 a,c	25.24±0.46 a,b,c	0.12±0.04 a	0.27±0.03	0.08±0.02	0.07±0.02 a,c	2.67±0.20 a,c	0.12±0.02 a
Ur L UAE	0.06±0.01 a	0.20±0.02 a,b,c	0.13±0.03 b	29.38±0.91	0.07±0.01	0.36±0.08	0.20±0.04 a,b	0.04±0.01	1.91±0.17	0.09±0.01
Ur L MH	0.05±0.00 a	0.09±0.01	0.13±0.01	31.83±0.88	0.08±0.01	0.35±0.03	0.14±0.01	0.05±0.01	1.68±0.12	0.11±0.01 a
Vo R UAE	0.03±0.01 c	0.03±0.00	0.08±0.01 b	31.23±2.17	0.04±0.01	0.22±0.06	0.07±0.01	0.02±0.00	1.12±0.13 b,c	0.05±0.02
Vo R MH	0.05±0.01 a	0.27±0.01 a,b,c	0.18±0.02	32.32±0.82	0.05±0.01	0.33±0.04	0.11±0.01	0.05±0.00	1.65±0.11	0.07±0.00

Table S1 – continuation. Effect of the foliar application of the botanical extracts on the volatile compounds profile (the amount of a single component calculated as a percentage (%) of the whole GC-MS chromatogram area) of cabbage head after harvest (N=3, mean ± SD).

Group	Heptadienal <2,4-trans, trans->	1,5-Hexanediol	Cymene <para->	Limonene	3-Octen-2-one	Benzeneacetaldehyde	2-Octenal	3-Octanol, 2,2-dimethyl-	Nonanal	Methyl methylthiomethyl disulfide	Allyl methyl trisulfide
RT, min	13.33	13.93	14.08	14.33	15.07	15.18	16.16	18.47	19.13	20.16	20.94
RI_exp	1011	1021	1025	1030	1041	1044	1052	1096	1099	1123	1127
RI_NIST	1012	1023	1025	1030	1039	1045	1060	1093	1104	1129	1135
RI_FFNSC	1013	1023	1025	1030	1036	1045	1059	1093	1104	1129	1134
RI_Adams	1007	1023	1024	1029	1035	1044	1054	1098	1100	1124	1132
C	0.15±0.01	0.06±0.00 c	0.17±0.01 c	0.22±0.01 b	0.03±0.00 b,c	0.03±0.00	0.34±0.04	0.14±0.01 c	0.24±0.01	0.82±0.03 b	0.12±0.01
CF	0.23±0.03	0.02±0.01 c	0.10±0.01 c	0.40±0.03 a,c	0.12±0.02 a,c	0.04±0.01	0.46±0.04 c	0.19±0.02	0.29±0.03	1.36±0.16 a,c	0.13±0.00
CB	0.14±0.02	0.15±0.02 a,b	0.34±0.01 a,b	0.12±0.02 b	0.08±0.01 a,b	0.06±0.00	0.25±0.03 b	0.28±0.04 a	0.27±0.04	0.76±0.04 b	0.14±0.01
Hp H UAE	0.20±0.02	0.06±0.01 c	0.28±0.06 b	0.36±0.02 a,c	0.07±0.01 b	0.07±0.01	0.42±0.01	0.25±0.03	0.32±0.04	1.04±0.09	0.12±0.03
Hp H MH	0.16±0.03	0.13±0.01 a,b	0.34±0.02 a,b	0.09±0.02 a,b	0.07±0.01 a,b	0.04±0.00	0.33±0.01	0.38±0.02 a,b	0.29±0.02	1.64±0.16 a,c	0.08±0.01
Sg L UAE	0.15±0.04	0.22±0.02 a,b,c	0.24±0.03 b	0.30±0.03 c	0.03±0.01 b,c	0.06±0.01	0.36±0.08	0.13±0.02 c	0.24±0.06	1.02±0.37	0.22±0.00 a,b
Sg L MH	0.23±0.03	0.06±0.01 c	0.15±0.01 c	0.14±0.01 b	0.08±0.01 a	0.04±0.00	0.50±0.05 c	0.23±0.04	0.28±0.00	1.23±0.15	0.14±0.03
To F UAE	0.29±0.02 a,c	0.03±0.00 c	0.23±0.02 b	0.22±0.02 b	0.06±0.01 b	0.05±0.00	0.46±0.11 c	0.31±0.06 a	0.39±0.01	1.22±0.10	0.32±0.06 a,b,c
To F MH	0.17±0.01	0.01±0.00 c	0.08±0.01 c	0.25±0.05 b,c	0.08±0.01 a	0.09±0.03 a,b	0.33±0.06	0.27±0.03 a	0.30±0.02	0.90±0.06	0.13±0.01
To L UAE	0.21±0.02	0.16±0.05 a,b	0.22±0.04 b,c	0.28±0.06 b,c	0.04±0.01 b	0.06±0.00	0.43±0.08	0.25±0.04	0.36±0.03	1.36±0.13 a,c	0.17±0.03
To L MH	0.33±0.07 a,c	0.24±0.03 a,b,c	0.11±0.02 c	0.20±0.01 b	0.11±0.02 a	0.08±0.01 a	0.71±0.09 a,b,c	0.36±0.03 a,b	0.48±0.08 a,b,c	1.06±0.06	0.33±0.05 a,b,c
Tp F UAE	0.22±0.04	0.10±0.00 b	0.18±0.05 c	0.25±0.02 b,c	0.11±0.02 a	0.04±0.01	0.48±0.03 c	0.31±0.01 a	0.45±0.09 a,c	1.00±0.07	0.16±0.03
Tp F MH	0.28±0.03 a,c	0.14±0.01 a,b	0.47±0.03 a,b,c	0.10±0.01 a,b	0.10±0.02 a	0.05±0.01	0.53±0.03 c	0.23±0.01	0.41±0.02 a	0.76±0.03 b	0.13±0.02
Ur L UAE	0.21±0.02	0.07±0.01 c	0.12±0.02 c	0.27±0.00 b,c	0.08±0.01 a	0.06±0.00	0.35±0.07	0.28±0.06 a	0.38±0.03	1.14±0.03	0.18±0.02
Ur L MH	0.26±0.02 c	0.03±0.01 c	0.18±0.02 c	0.56±0.05 a,b,c	0.06±0.01 b	0.04±0.00	0.37±0.01	0.20±0.02	0.24±0.04	0.94±0.08	0.18±0.01
Vo R UAE	0.18±0.00	0.03±0.00 c	0.23±0.04 b	0.15±0.03 b	0.04±0.01 b	0.03±0.00	0.38±0.05	0.17±0.02	0.19±0.02	0.85±0.04 b	0.05±0.01
Vo R MH	0.29±0.02 a,c	0.16±0.01 a,b	0.14±0.02 c	0.19±0.02 b	0.06±0.01 b	0.05±0.00	0.44±0.03	0.31±0.03 a	0.31±0.06	1.13±0.03	0.14±0.01

Table S1 – continuation. Effect of the foliar application of the botanical extracts on the volatile compounds profile (the amount of a single component calculated as a percentage (%) of the whole GC-MS chromatogram area) of cabbage head after harvest (N=3, mean ± SD).

Group	Non-(2*E*)-enal	2-Decanone	Disulfide, 1-methylethyl isopentyl	Tetrasulfide, dimethyl-	Nona-(2*E*, 4*E*)-dienal	Benzeneacetic acid, ethyl ester	Geraniol	Dec-(2E)-enal	Thiophene, 2-hexyl-	p-Menth-4(8)-en-9-ol	Indole
RT, min	22.69	24.96	25.27	25.94	26.22	27.82	29.26	29.43	30.05	30.52	31.31
RI_exp	1158	1194	1196	1213	1217	1238	1262	1263	1271	1279	1289
RI_NIST	1162	1193	1199	1217	1216	1244	1255	1263	1277	1284	1295
RI_FFNSC	1163	1191	1197	1214	1218	1243	1255	1265	1277	1284	1291
RI_Adams	1161	1192	1194	1211	1213	1244	1252	1263	1275	1289	1291
C	0.09±0.01	0.04±0.00	0.27±0.06 b,c	15.91±1.03	0.09±0.01	0.14±0.02	0.11±0.02	0.12±0.03	0.09±0.01	0.09±0.00	0.65±0.06 b,c
CF	0.16±0.01	0.07±0.01	0.11±0.01 a	13.54±0.23 c	0.18±0.02	0.11±0.01	0.06±0.01	0.20±0.03	0.09±0.02	0.16±0.00	0.35±0.05 a
CB	0.13±0.01	0.07±0.01	0.09±0.01 a	17.59±0.40 b	0.14±0.01	0.12±0.01	0.03±0.01	0.14±0.03	0.07±0.01	0.09±0.02	0.25±0.05 a
Hp H UAE	0.15±0.01	0.06±0.01	0.16±0.04	13.21±0.27 a,c	0.15±0.03	0.15±0.00	0.15±0.01 c	0.14±0.02	0.12±0.02	0.12±0.02	0.59±0.01 c
Hp H MH	0.11±0.02	0.04±0.01	0.12±0.01	20.41±0.76 a,b,c	0.19±0.04 a	0.21±0.04 b,c	0.12±0.01 c	0.15±0.04	0.08±0.01	0.10±0.02	0.32±0.04 a
Sg L UAE	0.15±0.03	0.04±0.01	0.19±0.00	15.31±0.94	0.10±0.03	0.15±0.02	0.23±0.01 a,b,c	0.16±0.03	0.08±0.03	0.12±0.02	0.57±0.10 c
Sg L MH	0.23±0.02 a,c	0.08±0.01 a	0.28±0.03 b,c	15.17±0.44	0.20±0.02 a	0.15±0.02	0.25±0.03 a,b,c	0.18±0.02	0.13±0.01	0.16±0.03	0.45±0.08
To F UAE	0.14±0.02	0.09±0.01 a	0.37±0.10 b,c	16.90±0.44 b	0.16±0.02	0.18±0.01 b	0.30±0.08 a,b,c	0.23±0.01 a	0.15±0.04 c	0.13±0.01	0.36±0.08 a
To F MH	0.13±0.01	0.10±0.01 a	0.29±0.01 b,c	13.73±0.35 c	0.17±0.05	0.12±0.02	0.05±0.01	0.16±0.01	0.10±0.02	0.10±0.02	0.23±0.07 a
To L UAE	0.16±0.01	0.10±0.01 a	0.19±0.00	13.31±0.64 a,c	0.18±0.04	0.12±0.02	0.22±0.02 a,b,c	0.17±0.02	0.33±0.01 a,b,c	0.10±0.01	0.55±0.08 c
To L MH	0.21±0.02 a,c	0.08±0.01	0.52±0.02 a,b,c	12.92±0.35 a,c	0.24±0.04 a,c	0.23±0.03 a,b,c	0.34±0.02 a,b,c	0.17±0.02	0.17±0.02 a,b,c	0.21±0.05 a,c	0.80±0.08 b,c
Tp F UAE	0.19±0.03 a	0.07±0.01	0.46±0.07 a,b,c	15.00±1.32 c	0.18±0.01	0.14±0.02	0.26±0.02 a,b,c	0.30±0.04 a,c	0.09±0.01	0.14±0.02	0.37±0.05 a
Tp F MH	0.25±0.03 a,b,c	0.15±0.02 a,b,c	0.26±0.05 c	11.95±0.51 a,c	0.22±0.04 a	0.17±0.03	0.07±0.00	0.24±0.04 a,c	0.15±0.01 c	0.14±0.00	0.36±0.08 a
Ur L UAE	0.14±0.03	0.05±0.01	0.34±0.06 b,c	16.41±0.48 b	0.16±0.01	0.19±0.02 b	0.22±0.02 a,b,c	0.20±0.01	0.13±0.02	0.08±0.03	0.48±0.07
Ur L MH	0.22±0.02 a,c	0.06±0.00	0.15±0.01	14.89±0.34 c	0.18±0.02	0.15±0.02	0.10±0.01	0.18±0.03	0.07±0.02	0.13±0.02	0.34±0.05 a
Vo R UAE	0.12±0.02	0.05±0.01	0.18±0.06	13.46±1.10 c	0.13±0.01	0.12±0.02	0.02±0.00	0.09±0.03 b	0.07±0.01	0.10±0.03	0.46±0.05
Vo R MH	0.16±0.02	0.09±0.01 a	0.20±0.01	16.96±0.34 b	0.17±0.01	0.15±0.01	0.18±0.02 b,c	0.16±0.01	0.12±0.02	0.13±0.04	0.45±0.12

Table S1 – continuation. Effect of the foliar application of the botanical extracts on the volatile compounds profile (the amount of a single component calculated as a percentage (%) of the whole GC-MS chromatogram area) of cabbage head after harvest (N=3, mean ± SD).

Group	IS	Deca-(2*E*, 4*E*)-dienal	Undec-(8*Z*)-enal	Un-decanol	Disul-fide, bu-tyl pen-tyl	5-Tride-canol	1-Tride-canol	Citronel-lyl val-erate	Butyli-dene phthal-ide	1-Penta-decanal	4-Hexa-decanol
RT, min	31.68	32.94	36.05	37.00	39.92	42.75	47.42	51.61	53.62	54.88	55.77
RI_exp	1297	1318	1365	1377	1420	1473	1551	1628	1675	1719	1769
RI_NIST	1294	1317	1370	1371	1414	1478	1568	1625	1674	1715	1774
RI_FFNSC	1294	1322	1365	1379	1417	1476	1556	1624	1673	1716	1774
RI_Adams	1294	1316	1360	1380	1418	1477	1561	1625	1672	1714	1769
C	42.92±0.35 b	0.49±0.13	0.13±0.01	0.10±0.02	0.06±0.00	0.09±0.01	0.05±0.01 b	0.15±0.01	0.07±0.01 b,c	0.33±0.04	0.04±0.01 c
CF	38.58±0.47 a	1.06±0.29	0.16±0.02	0.14±0.04	0.01±0.00	0.07±0.02	0.10±0.01 a	0.19±0.01	0.03±0.00 a	0.54±0.04	0.05±0.01 c
CB	39.70±0.95	0.67±0.03	0.18±0.03	0.07±0.01	0.05±0.01	0.15±0.01	0.10±0.02	0.18±0.04	0.01±0.00 a	0.52±0.06	0.12±0.03 a,b
Hp H UAE	41.95±0.63	0.49±0.12	0.12±0.02	0.11±0.01	0.08±0.01 b	0.11±0.01	0.08±0.01	0.16±0.02	0.02±0.01 a	0.55±0.16	0.05±0.01 c
Hp H MH	32.41±0.40 a,b,c	0.84±0.12	0.17±0.02	0.11±0.01	0.02±0.01	0.13±0.00	0.07±0.01	0.10±0.01	0.01±0.00 a	0.52±0.05	0.05±0.01 c
Sg L UAE	45.81±1.46 b,c	0.64±0.17	0.17±0.02	0.11±0.03	0.09±0.00 b	0.01±0.00 c	0.08±0.01	0.18±0.02	0.06±0.01 b,c	0.47±0.06	0.10±0.02 a,b
Sg L MH	37.26±0.63 a	1.36±0.18 a,c	0.20±0.05	0.10±0.02	0.11±0.01 b	0.14±0.02	0.09±0.01	0.32±0.04 a,b,c	0.02±0.00 a	1.06±0.11 a,b,c	0.08±0.00
To F UAE	32.16±1.12 a,b,c	1.20±0.15 a	0.26±0.03 a,b	0.14±0.01	0.15±0.02 a,b,c	0.19±0.05 a,b	0.14±0.02 a	0.17±0.04	0.04±0.01 a,c	0.36±0.03	0.08±0.01
To F MH	43.25±0.35 b	0.92±0.04	0.27±0.04 a,b	0.12±0.02	0.09±0.01 b	0.10±0.02	0.10±0.01 a	0.22±0.01	0.02±0.00 a	0.95±0.15 a,b,c	0.07±0.01
To L UAE	45.48±1.17 b,c	0.96±0.07	0.18±0.01	0.10±0.02	0.11±0.02 b	0.13±0.01	0.14±0.02 a	0.10±0.02	0.09±0.02 b,c	0.40±0.02	0.10±0.01 a,b
To L MH	33.17±1.03 a,b,c	1.53±0.14 a,c	0.21±0.02	0.15±0.02 c	0.16±0.00 a,b,c	0.17±0.04 b	0.11±0.01 a	0.40±0.04 a,b,c	0.01±0.01 a	1.03±0.05 a,b,c	0.12±0.02 a,b
Tp F UAE	34.11±2.06 a,b,c	1.11±0.13 a	0.24±0.04 a	0.19±0.03 a,c	0.15±0.01 a,b,c	0.19±0.05 a,b	0.06±0.01	0.19±0.02	0.09±0.01 b,c	0.28±0.06	0.12±0.02 a,b
Tp F MH	43.95±0.63 b,c	1.39±0.09 a,c	0.31±0.04 a,b,c	0.17±0.02 c	0.10±0.02 b	0.11±0.01	0.09±0.00	0.21±0.02	0.03±0.00 a,c	0.55±0.06	0.07±0.01
Ur L UAE	38.02±0.71 a	0.79±0.15	0.29±0.02 a,b,c	0.15±0.02 c	0.11±0.02 b	0.20±0.03 a,b	0.08±0.01	0.17±0.02	0.01±0.00 a	0.28±0.06	0.10±0.01 a
Ur L MH	37.67±0.23 a	1.17±0.08 a	0.18±0.03	0.15±0.00 c	0.03±0.01	0.13±0.02	0.05±0.00 b	0.15±0.01	0.03±0.00 a,c	0.45±0.09	0.06±0.00 c
Vo R UAE	43.59±0.82 b,c	1.10±0.34 a	0.12±0.02	0.05±0.01 b	0.12±0.04 a,b,c	0.10±0.02	0.08±0.02	0.21±0.03	0.01±0.00 a	1.31±0.09 a,b,c	0.07±0.01 c
Vo R MH	34.00±1.29 a,b,c	1.19±0.15 a	0.17±0.01	0.12±0.02	0.07±0.00 b	0.12±0.01	0.11±0.01 a	0.32±0.04 a,b,c	0.02±0.00 a	0.79±0.11 a	0.05±0.02 c

Table S2. Effect of the foliar application of the botanical extracts on the fatty acids composition (the amount of a single component calculated as a percentage (%) of the whole GC-MS chromatogram area) of cabbage head after harvest (N=3, mean ± SD).

**Group**	**C12:0 Dodeca-noic acid, methyl ester**	**C14:0 Tetradeca-noic acid, methyl ester**	**C14:0 Tetradeca-noic acid, ethyl ester**	**C15:0 Pentadeca-noic acid, methyl ester**	**C18:1 Z-6-Octa-decenoic acid, methyl ester**	**C16:0 ω–3 Hexa-decanoic acid, methyl ester**	**C16:1 ω–7 Z-9-Hexadecenoic acid, methyl es-[illegible]**	**C17:0-iso Hexadeca-noic acid, 15-methyl-, methyl ester**	**C17:0-anteiso Hexadeca-noic acid, 14-[illegible]**	**C17:0 Heptade-canoic acid, methyl ester**
RT, min	18.725	24.210	26.020	26.825	27.330	29.445	30.360	30.685	31.060	31.795
RI_exp	1200	1400	1470	1500	1519	1600	1640	1653	1669	1701
RI_lit	1203	1402	1471	1503	1523	1600	1632	1656	1693	1700
C	0.08±0.01 b,c	0.51±0.02	0.05±0.01 c	0.62±0.02 c	0.09±0.01 b,c	29.30±0.14 b,c	0.41±0.02	0.14±0.01 c	0.95±0.03 b,c	0.40±0.01 b,c
CF	0.20±0.01 a,c	0.63±0.03 c	0.11±0.00 c	0.73±0.04	0.19±0.02 a,c	34.54±0.20 a	0.33±0.03	0.12±0.01 c	0.74±0.03 a,c	0.19±0.02 a,c
CB	0.41±0.02 a,b	0.50±0.02 b	0.21±0.02 a,b	0.83±0.03 a	0.29±0.02 a,b	35.47±0.11 a	0.39±0.02	0.25±0.02 a,b	0.56±0.02 a,b	0.55±0.02 a,b
Hp H UAE	0.15±0.01 c	0.47±0.04 b	0.09±0.01 c	0.53±0.04 b,c	0.10±0.00 b,c	30.47±0.42 a,b,c	0.31±0.02 a	0.15±0.02 c	0.51±0.04 a,b	0.29±0.02 a,b,c
Hp H MH	0.23±0.02 a,c	0.66±0.03 a,c	0.24±0.02 a,b,c	0.86±0.02 a,b	0.24±0.02 a	32.44±0.24 a,c	0.24±0.02 a,c	0.52±0.03 a,b,c	0.72±0.03 a,c	0.19±0.02 a,c
Sg L UAE	0.16±0.01 c	0.41±0.02 b	0.10±0.00 c	0.66±0.03 b,c	0.08±0.01 b,c	25.53±0.18 a,b,c	0.41±0.03	0.13±0.01 c	0.62±0.03 a	0.13±0.01 a,c
Sg L MH	0.39±0.05 a,b	0.81±0.05 a,b,c	0.12±0.01 a,c	0.71±0.03 b	0.10±0.01 b,c	33.04±0.23 a,c	0.45±0.03 b	0.35±0.03 a,b,c	0.45±0.03 a,b	0.59±0.02 a,b
To F UAE	0.51±0.02 a,b,c	0.56±0.06	0.11±0.02 a,c	0.57±0.04 b,c	0.13±0.01 c	29.48±0.23 b,c	0.38±0.02	0.21±0.02 b	0.59±0.03 a,b	0.56±0.02 a,b
To F MH	0.66±0.02 a,b,c	0.79±0.03 a,b,c	0.25±0.03 a,b,c	0.99±0.04 a,b,c	0.31±0.02 a,b	37.48±0.30 a,b,c	0.28±0.01 a,c	0.37±0.02 a,b,c	0.52±0.03 a,b	0.54±0.03 a,b
To L UAE	0.51±0.02 a,b,c	1.28±0.05 a,b,c	0.11±0.01 c	0.72±0.04 b	0.12±0.01 b,c	24.92±0.15 a,b,c	0.52±0.03 a,b,c	0.37±0.02 a,b,c	0.44±0.03 a,b	0.47±0.03 b
To L MH	0.43±0.03 a,b	0.95±0.03 a,b,c	0.21±0.02 a,b,c	0.86±0.03 a,b	0.17±0.01 a,c	39.47±0.34 a,b,c	0.57±0.02 a,b,c	0.23±0.03 b	0.82±0.05 a,c	0.48±0.02 b
Tp F UAE	0.23±0.03 a,c	0.56±0.02	0.13±0.01 a,c	0.51±0.02 b,c	0.37±0.04 a,b,c	28.99±0.18 b,c	0.37±0.03	0.20±0.02	0.71±0.03 a,c	0.35±0.03 b,c
Tp F MH	0.34±0.04 a,b	0.83±0.03 a,b,c	0.13±0.01 a,c	0.70±0.06 b	0.24±0.02 a	35.94±0.13 a,b	0.34±0.02	0.19±0.02	0.93±0.03 b,c	0.44±0.03 b,c
Ur L UAE	0.20±0.03 a,c	0.40±0.02 b	0.17±0.01 a,b,c	0.60±0.02 b,c	0.11±0.01 b,c	26.05±0.17 a,b,c	0.41±0.03	0.22±0.03 b	0.52±0.02 a,b	0.34±0.03 b,c
Ur L MH	0.13±0.01 c	0.66±0.02 a,c	0.05±0.00 b,c	0.74±0.04 b	0.08±0.01 b,c	32.55±0.21 a,c	0.43±0.04 b	0.17±0.04	0.77±0.03 a,c	0.24±0.02 a,c
Vo R UAE	0.24±0.02 a,c	0.79±0.02 a,b,c	0.08±0.01 c	0.52±0.04 b,c	0.11±0.01 b,c	23.02±0.33 a,b,c	0.41±0.02	0.22±0.02 b	0.52±0.03 a,b	0.14±0.01 a,c
Vo R MH	0.50±0.01 a,b,c	0.61±0.02	0.15±0.02 a,c	0.84±0.03 a,b	0.09±0.01 b,c	32.31±0.49 a,b,c	0.58±0.04 a,b,c	0.33±0.02 a,b	0.64±0.04 a	0.77±0.04 a,b,c

Statistically significant differences ($p < 0.05$) (a) between the control group (C) and the botanical extracts; (b) between the formulation (CF) and the botanical extracts; (c) between commercial biostimulant (CB) and the botanical extracts; Hp H, *Hypericum perforatum* L. (St. John's wort, herb); Sg L, *Solidago gigantea* Ait. (giant goldenrod, leaf); To F, To L, *Taraxacum officinale* (L.) Weber ex F.H. Wigg (common dandelion, flower, leaf); Tp F, *Trifolium pratense* L. (red clover, flower); Ur L, *Urtica dioica* L. (nettle, leaf); Vo R, *Valeriana officinalis* L. (valerian, root).

Table S2 – continuation. Effect of the foliar application of the botanical extracts on the fatty acids composition (the amount of a single component calculated as a percentage (%) of the whole GC-MS chromatogram area) of cabbage head after harvest (N=3, mean ± SD).

Group	C18:0 Heptadecanoic acid, 16-methyl-, methyl ester	C18:0 ω–3 Octadecanoic acid, methyl ester	C18:1 ω–9 9Z-9-Octadecenoic acid, ethyl ester	C18:0 Octadecanoic acid, ethyl ester	C18:2 ω–6 Linoleic acid, methyl ester	C17:1 11-Hexadecenoic acid, 15-methyl-, methyl	C18:3 ω–3 Linolenic acid, methyl ester	C20:0 Eicosanoic acid, methyl ester	C18:2 ω–6 9,12-Octadecadienoic acid, methyl ester	C22:0 Docosanoic acid, methyl ester	C24:0 Tetracosanoic acid, methyl ester
RT, min	33.035	34.190	34.865	35.740	36.275	36.970	37.705	38.035	39.335	40.630	42.615
RI_exp	1755	1797	1839	1877	1902	1931	1986	1998	2095	2195	2395
RI_lit	1753	1800	1847	1873	1893	1925	1992	2000	2093	2200	2400
C	0.11±0.02 b	7.78±0.08 c	8.83±0.12 b	0.62±0.03	22.71±0.13 b,c	1.58±0.05 b,c	22.25±0.09 b,c	1.47±0.06 b,c	0.15±0.02	0.92±0.02 c	1.00±0.05 b
CF	0.42±0.02 a,c	7.46±0.10 c	9.27±0.15 a,c	0.54±0.05	20.18±0.23 a,c	0.41±0.01 a,c	18.71±0.23 a,c	2.01±0.05 a,c	0.21±0.02	0.98±0.02 c	2.04±0.04 a,c
CB	0.19±0.02 b	5.73±0.12 a,b	8.44±0.09 b	0.58±0.03	21.19±0.11 a,b	0.73±0.03 a,b	19.42±0.12 a,b	2.40±0.05 a,b	0.10±0.01	0.73±0.02 a,b	1.02±0.03 b
Hp H UAE	0.10±0.01 b,c	8.33±0.18 a,b,c	8.80±0.07 c	0.37±0.04 a,b,c	22.28±0.21 b,c	1.28±0.03 a,b,c	22.66±0.24 b,c	1.22±0.04 a,b,c	0.12±0.01	0.89±0.03 c	0.88±0.03 b
Hp H MH	0.13±0.01 b	6.76±0.07 a,b,c	9.54±0.09 a,b,c	0.72±0.05 b,c	20.69±0.29 a,b	1.99±0.04 a,b,c	18.24±0.16 a,b,c	1.86±0.05 a,c	1.04±0.04 a,b,c	1.11±0.05 a,b,c	1.57±0.09 a,b,c
Sg L UAE	0.10±0.01 b,c	3.66±0.05 a,b,c	7.65±0.09 a,b,c	0.29±0.02 a,b,c	25.78±0.17 a,b,c	1.00±0.09 a,b,c	30.97±0.12 a,b,c	0.97±0.05 a,b,c	0.14±0.01	0.46±0.05 a,b,c	0.76±0.03 a,b,c
Sg L MH	0.26±0.03 a,b	5.16±0.08 a,b,c	11.09±0.10 a,b,c	0.40±0.03 a,c	18.42±0.37 a,c	2.89±0.04 a,b,c	16.68±0.20 a,c	2.95±0.05 a,b,c	1.47±0.05 a,b,c	1.68±0.02 a,b,c	1.96±0.04 a,c
To F UAE	0.07±0.01 b,c	4.17±0.02 a,b,c	7.55±0.11 a,b,c	0.32±0.02 a,b,c	23.62±0.16 a,b,c	0.78±0.04 a,b	23.83±0.10 a,b,c	2.51±0.10 a,b	0.54±0.08 a,b,c	1.04±0.08 c	2.46±0.10 a,b,c
To F MH	0.34±0.02 a,c	8.87±0.05 a,b,c	6.75±0.05 a,b,c	0.63±0.03	18.59±0.13 a,c	0.80±0.03 a,b	16.53±0.21 a,b,c	2.05±0.05 a,b,c	0.32±0.02 a,c	1.12±0.02 a,b,c	1.80±0.03 a,c
To L UAE	0.43±0.04 a,c	3.10±0.07 a,b,c	6.09±0.10 a,b,c	0.19±0.02 a,b,c	22.69±0.14 b,c	0.52±0.02 a,c	34.56±0.21 a,b,c	1.12±0.06 a,b,c	0.30±0.02 a,c	0.53±0.04 a,b,c	1.02±0.05 b
To L MH	0.38±0.02 a,c	7.35±0.03 a,c	12.68±0.12 a,b,c	0.82±0.02 a,b,c	15.38±0.53 a,b,c	0.91±0.03 a,b,c	11.16±0.07 a,b,c	2.98±0.06 a,b,c	0.37±0.02 a,b,c	1.71±0.04 a,b,c	2.06±0.05 a,c

Tp F UAE	0.06±0.01 b,c	6.12±0.09 a,b,c	7.46±0.06 a,b,c	0.54±0.03	22.68±0.28 b,c	1.35±0.05 a,b,c	25.02±0.07 a,b,c	1.36±0.06 b,c	0.09±0.01	1.24±0.05 a,b,c	1.64±0.11 a,b,c
Tp F MH	0.22±0.03 a,b	6.50±0.11 a,b,c	7.87±0.05 a,b,c	0.67±0.03 b	19.56±0.08 a,c	1.67±0.08 b,c	17.52±0.09 a,c	1.77±0.03 a,c	1.36±0.03 a,b,c	1.03±0.04 c	1.75±0.03 a,b,c
Ur L UAE	0.20±0.03 a,b	3.64±0.10 a,b,c	8.20±0.13 a,b	0.56±0.08	25.18±0.14 a,b,c	1.12±0.03 a,b,c	28.73±0.27 a,b,c	1.34±0.05 b,c	0.13±0.02	0.79±0.06	1.07±0.11 b
Ur L MH	0.10±0.01 b,c	5.14±0.09 a,b,c	7.74±0.07 a,b,c	0.54±0.02	22.16±0.15 b,c	1.31±0.03 a,b,c	22.28±0.18 b,c	1.52±0.05 b,c	1.07±0.04 a,b,c	0.71±0.06 a,b	1.63±0.09 a,b,c
Vo R UAE	0.26±0.02 a,b	2.72±0.08 a,b,c	5.23±0.05 a,b,c	0.39±0.01 a,b,c	25.91±0.21 a,b,c	0.63±0.02 a,b	36.93±0.13 a,b,c	0.72±0.03 a,b,c	0.33±0.02 a,c	0.38±0.02 a,b,c	0.44±0.04 a,b,c
VoR MH	0.36±0.03 a,c	6.18±0.02 a,b,c	12.32±0.24 a,b,c	0.77±0.04 a,b,c	17.55±0.32 a,b,c	2.82±0.07 a,b,c	15.29±0.13 a,b,c	2.00±0.08 a,b,c	1.73±0.10 a,b,c	1.50±0.07 a,b,c	2.68±0.05 a,b,c
